# Rare human Caspase-6-R65W and Caspase-6-G66R variants identify a novel regulatory region of Caspase-6 activity

**DOI:** 10.1038/s41598-018-22283-z

**Published:** 2018-03-13

**Authors:** Agne Tubeleviciute-Aydin, Libin Zhou, Gyanesh Sharma, Ishankumar V. Soni, Sergey N. Savinov, Jeanne A. Hardy, Andrea C. LeBlanc

**Affiliations:** 1 Bloomfield Center for Research in Aging, Lady Davis Institute for Medical Research, Jewish General Hospital, 3755 Ch. Cote Ste-Catherine, Montreal, QC H3T 1E2 Canada; 2 Department of Neurology and Neurosurgery, McGill University, 3775 University St., Montreal, QC H3A 2B4 Canada; 3 Department of Anatomy and Cell Biology, McGill University, 3640 University St., Montreal, QC H3A 0C7 Canada; 4 Department of Chemistry, University of Massachusetts Amherst, 710 N Pleasant St., Amherst, MA 01003 USA; 5 Department of Biochemistry and Molecular Biology, University of Massachusetts Amherst, 240 Thatcher Way, Amherst, MA 01003 USA

## Abstract

The cysteine protease Caspase-6 (Casp6) is a potential therapeutic target of Alzheimer Disease (AD) and age-dependent cognitive impairment. To assess if Casp6 is essential to human health, we investigated the effect of *CASP6* variants sequenced from healthy humans on Casp6 activity. Here, we report the effects of two rare Casp6 amino acid polymorphisms, R65W and G66R, on the catalytic function and structure of Casp6. The G66R substitution eliminated and R65W substitution significantly reduced Casp6 catalytic activity through impaired substrate binding. In contrast to wild-type Casp6, both Casp6 variants were unstable and inactive in transfected mammalian cells. In addition, Casp6-G66R acted as a dominant negative inhibitor of wild-type Casp6. The R65W and G66R substitutions caused perturbations in substrate recognition and active site organization as revealed by molecular dynamics simulations. Our results suggest that full Casp6 activity may not be essential for healthy humans and support the use of Casp6 inhibitors against Casp6-dependent neurodegeneration in age-dependent cognitive impairment and AD. Furthermore, this work illustrates that studying natural single amino acid polymorphisms of enzyme drug targets is a promising approach to uncover previously uncharacterized regulatory sites important for enzyme activity.

## Introduction

Caspase-6 (Casp6) is a member of the caspase family of cysteinyl proteases involved in regulated cell death and control of inflammatory and immune responses^1^. Casp6 has been classified as an apoptotic executioner caspase due to its short pro-domain and its homology to Caspase-3 (Casp3) and Caspase-7^2^. However, Casp6 harbours many features that distinguish it from the executioner caspases. Casp6 initiates apoptosis in certain cell types by activating Caspase-8^3^, adopts unique conformations relative to other caspases^4,5^, self-activates intramolecularly^6^, does not induce cell death when solely activated in mammalian cell lines^7,8^ and has distinct substrate specificity^9^.

Casp6 is an attractive target for rational drug design against age-dependent cognitive impairment and AD. Casp6 has been strongly associated with AD^10,11^. Casp6 is present in neuritic plaque, neuropil thread and neurofibrillary tangle pathological lesions in sporadic and familial AD brains^11,12^. Higher Casp6 activity in brains correlates with and predicts a lower performance in episodic memory in aged human individuals^13,14^. In transgenic mice, human Casp6 activation in the hippocampal CA1 region is sufficient to cause neurodegeneration, inflammation and age-dependent memory impairment^15^. Casp6 activity is involved in axonal degeneration of nerve growth factor-deprived mouse sensory neurons^16–18^ and in serum-deprived or amyloid precursor protein-transfected primary human CNS neurons^19^. Casp6 cleaves α-Tubulin, microtubule-associated Tau protein and actin-regulating post-synaptic density proteins, Drebrin, Spinophilin and α-Actinin-1 and -4^20,21^. Therefore, Casp6 likely deregulates the neuronal cytoskeleton through its proteolytic activity. Furthermore, Casp6 cleaves the valosin-containing protein (VCP) and impairs its role in the ubiquitin proteasome system-mediated misfolded protein degradation pathway^22^. Recently, a quantitative assessment of substrate preference for Casp6 has identified protein substrates involved in the regulation of transcription, cell cycle, cell death, RNA splicing, cytoskeleton and the DNA damage response in Jurkat cells, thus revealing abundant protein substrates for Casp6 in human cells^9^. Nevertheless, Casp6 is barely detectable in human foetal and aged brain^23^. While the Casp6 null mouse is relatively normal^24^, the absence of Casp6 is associated with enhanced differentiation of B cells into plasma cells and increased production of antibodies^25^. A recent novel null Casp6 mouse possibly expressing a short catalytically inactive form of Casp6, revealed increased cortical and striatal brain volumes and age-dependent learning deficits^26^.

Casp6 is expressed as an inactive dimeric zymogen (proCasp6) composed of a short pro-domain (Pro), a large subunit (LS) containing the active site cysteine-histidine catalytic dyad, an inter-subunit linker (L) and a small subunit (SS). The Casp6 zymogen is processed intramolecularly at TEVD_193_^6^ and intermolecularly^27^ at TETD_23_, DVVD_179_ and TEVD_193_, resulting in the release of LS and SS and dimeric re-assembly of LS/SS homodimers to generate the active Casp6^28^. The overall structure of Casp6^5,28^ is similar to that of human Caspase-1, -2, -3, -7, -8 and -9^5,29–34^. The ligand-free Casp6, however, differs from other caspases by the presence of 60’s and 90’s extended helices flanking the Casp6 active site^4,28^. Caspases cleave their substrates mostly at an aspartate, glutamate or phosphoserine residues^35^. Caspases catalyse the cleavage of amide bonds via nucleophilic attack of the cysteine thiolate (Cys163 in Casp6) at the substrate amide carbonyl. During catalysis, the histidine (His121 in Casp6) activates the catalytic cysteine^36^. Because of the high conservation of active sites throughout the caspase family, development of selective caspase active site inhibitors has proven to be challenging^37,38^.

Currently, targeting allosteric sites is recognized as a more viable method for designing selective inhibitors for caspases, including Casp6^4^. Different methods, including surface plasmon resonance with small molecules^39^, phage display-based peptide library^40^ and small molecule^41^ screening against Casp6 zymogen or active Casp6, have identified allosteric sites regulating Casp6 activity and/or proCasp6 activation. Furthermore, Casp6 phosphorylation at serine 257 and zinc binding at lysine 36, glutamine 244 and histidine 287 allosterically regulate Casp6 activity^42–44^. In contrast to the traditional approach using general site-directed mutagenesis, here, rare *CASP6* non-synonymous single nucleotide polymorphisms (SNPs) resulting in missense single amino acid changes in human Casp6 were analysed to uncover potential regulatory sites of Casp6 evolved by nature. Our results show that two Casp6 variants, Casp6-G66R and Casp6-R65W, have less self-processing and exogenous proteolytic activity than Casp6-WT and reveal a novel regulatory area of Casp6 activity. Furthermore, the existence of these Casp6 variants suggests that full Casp6 activity may be dispensable in humans.

## Results

### Identification of rare missense variants of the human *CASP6 gene* with altered activity

**Figure 1:**
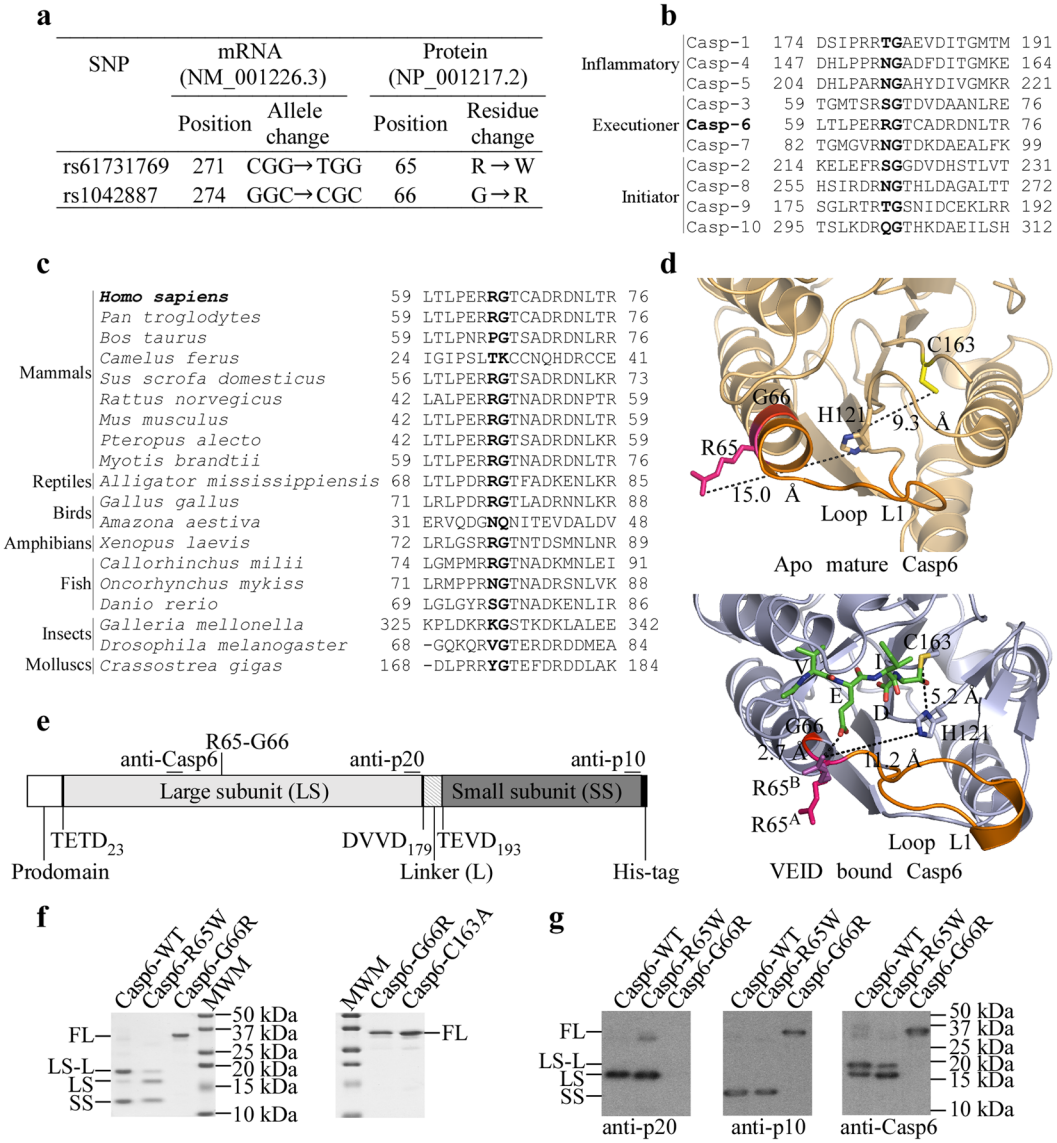
Identification of rare human *CASP6* variants and self-processing of purified recombinant Casp6. (**a**) *CASP6* single nucleotide polymorphisms encoding amino acid substitutions at codon 65 and 66 of the Casp6 protein identified in the NCBI SNP database. (**b** and **c**) Multiple sequence alignment of the sequence encoding and flanking amino acids 65 and 66 of Casp6 with other human caspases (**b**) and with Casp6 from various organisms (**c**). Human Casp6 R65 and G66 are shown in bold. (**d**) Pymol structures derived from 2WDP chain D^28^ and 3OD5 chain A^6^ indicating the location of R65 (pink) and G66 (red) in the crystal structure of apo form (light orange; top panel) and Ac-VEID-CHO (green) bound form (light blue; bottom panel) of human mature Casp6. The position of R65 side chain from 3OD5 chain B is depicted in magenta (R65^B^) and is superimposed on the structure of 3OD5 chain A, which shows R65 position in pink (R65^A^). The broken lines indicate distances between amino acid residues. (**e**) Schematic representation of full-length recombinant C-terminally His-tagged proCasp6 and its domains, the position of the TETD_23_, DVVD_179_ and TEVD_193_ cleavage sites and the epitopes recognized by Casp6 antibodies. (**f**) Coomassie stain of SDS-PAGE with ~2 µg of purified recombinant Casp6-WT, Casp6-R65W, Casp6-G66R and catalytically inactive Casp6-C163A showing full length (FL), large subunit with (LS-L) and without linker (LS) and small subunit (SS) of Casp6. (**g**) Western-blot analyses of purified recombinant Casp6-WT, Casp6-R65W and Casp6-G66R with Casp6 Pharmingen anti-p10, neoepitope 10630 anti-p20 and Santa Cruz anti-Casp6 (LS) antibodies or antiserum. Full-length images of gels and blots are provided in Supplementary Information.

Since Casp6 polymorphisms have not yet been associated with inherited diseases, we reasoned that natural polymorphisms of human Casp6 could inhibit its activity without revealing an obvious phenotype in humans. We screened the NCBI PubMed SNP database and identified several missense variants of *CASP6*. In this paper, we focus on two adjacent polymorphisms, Casp6-R65W (ExAC aggregated population allele frequency 0.0001159) and Casp6-G66R (no frequency data available) (Fig. 1a). Interestingly, despite being next to each other, R65 is found only in Casp6 compared to nine other human caspases, whereas G66 is entirely conserved in human caspases (Fig. 1b). Casp6-R65 is present in most mammals and some reptiles, birds and amphibians, but varies in fish, insects and molluscs (Fig. 1c). Despite these differences, no W65 was detected in the 19 species examined. By contrast, G66 is conserved in all species except for the *Camelus ferus* mammal and the *Amazona aestiva* bird. R65 and G66 are located in the extended helix B (amino acids 61-80), also known as the 60’s helix of apo mature Casp6 (Fig. 1d). In the apo form, R65 and G66 are close to the active site but the two side-chain moieties are not interacting with other amino acid residues of Casp6 and the side chain of R65 is pointing away from the Casp6 active site catalytic dyad H121-C163. In a catalytically competent form of Casp6, represented by covalently bound Ac-VEID-CHO-Casp6, the 60’s helix undergoes partial unfolding into the extension of L1 loop. G66 is retained in a position similar to that of the apo form, whereas R65 is solvent exposed and is observed in different conformations in two protomers of the dimeric structure (PDB ID: 3OD5): it either points away from the Casp6 active site (chain A) as in the apo form or is located close (2.7 Å) to the glutamate residue at the P3 position of Ac-VEID-CHO (chain B). Prokaryotically expressed recombinant Casp6-WT efficiently self-processes at TEVD_193,_ thus resulting in the generation of a higher amount of the large subunit with linker (LS-L) than without linker (LS) (Fig. 1e and f). By contrast, recombinant Casp6-R65W generates more of the LS than LS-L, whereas Casp6-G66R remains unprocessed and migrates with the catalytically inactive proCasp6-C163A mutant (Fig. 1f). The identity of the subunits was confirmed by western blot analyses with neoepitope antiserum against the LS cleaved at DVVD_179,_ anti-p10 antibody that detects both full length (FL) Casp6 and its small subunit (SS) and anti-Casp6 antibody that detects LS, LS-L, or FL Casp6 (Fig. 1g). These results indicate that the auto-processing activity of these two rare human variants of Casp6 significantly differ from Casp6-WT

### Casp6-R65W and Casp6-G66R display reduced proteolytic activity on peptide and protein substrates through impaired active site binding

**Figure 2:**
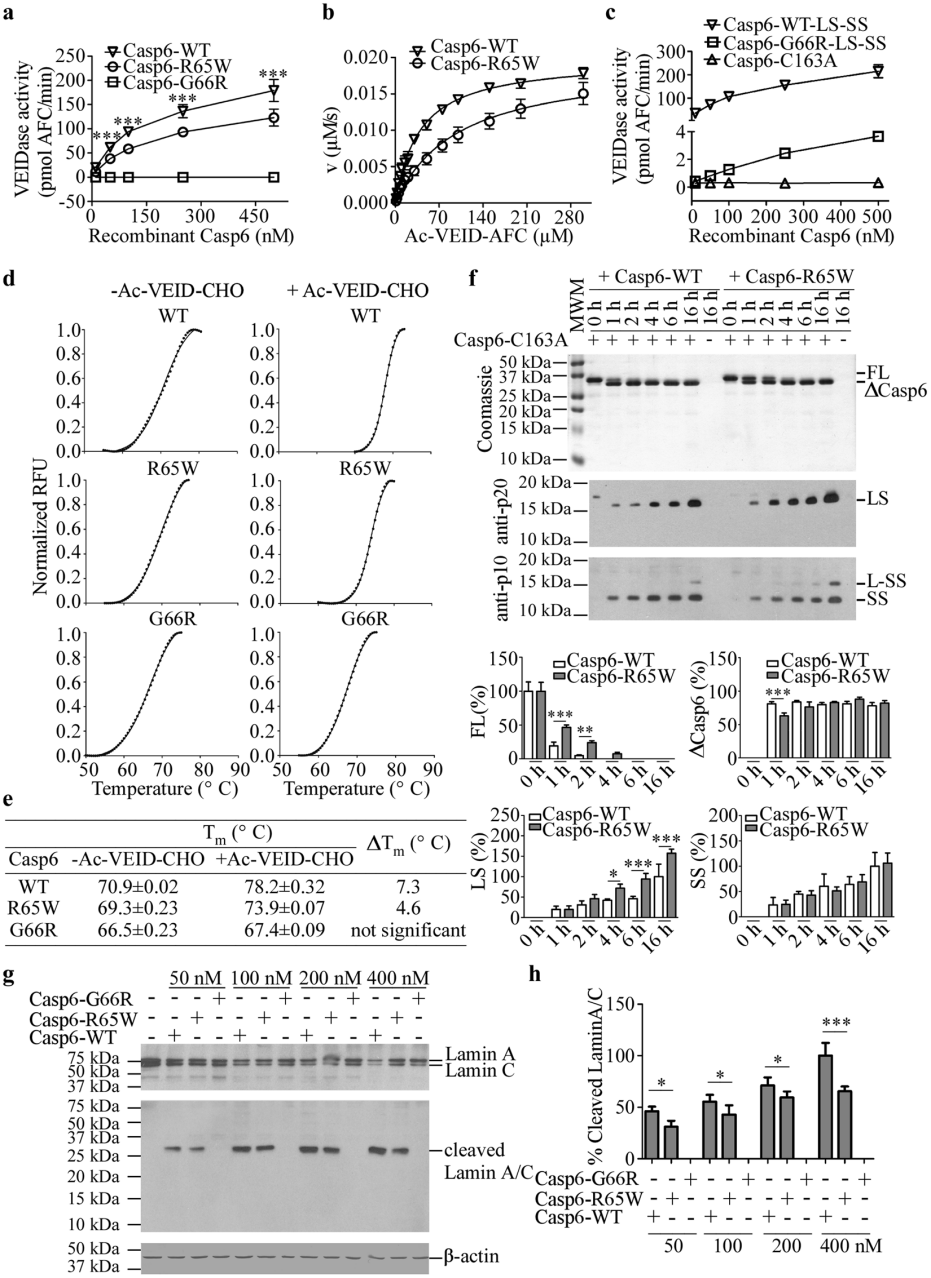
Casp6 activity on peptide and protein substrates and thermal stability. (**a**) Casp6 activity of 10–500 nM active site titrated recombinant Casp6-WT, Casp6-R65W and Casp6-G66R on Ac-VEID-AFC. Data represent mean ± SD from three independent experiments. One-way ANOVA (p < 0.0001) followed by Bonferroni’s multiple comparison tests compares Casp6-R65W with Casp6-WT, ***p < 0.001 (**b**) Relationship between initial reaction velocity (v) and substrate concentration of Casp6-WT and Casp6-R65W catalysed Ac-VEID-AFC cleavage. Data were fitted into Michaelis-Menten equation using nonlinear regression and represent the mean ± SD from three independent experiments. (**c**) Activity of co-expressed LS and SS from Casp6-G66R and Casp6-WT. Data represent mean ± SD from three independent experiments. (**d**) The thermal melting curves of Casp6-WT, Cap6-R65W and Casp6-G66R with and without Ac-VEID-CHO. (**e**) The thermal melting temperatures (T_m_) defined as the temperature at the midpoint of the unfolding response in (d). The difference between the melting temperatures of the unliganded and Ac-VEID-CHO-bound protein were calculated as ΔT_m_ = T_m(with Ac-VEID-CHO)_ − T_m(without Ac−VEID-CHO)_. Each experiment was done in triplicates using three different aliquots of caspase-6 variants on three separate days. (**f**) Coomassie gel (top panel) and neoepitope anti-p20 (middle panel) and anti-p10 (bottom panel) western blots of time-dependent cleavage of Casp6-C163A by active Casp6-WT or Casp6-R65W. Quantitation of specific protein or peptide bands expressed as a % of time 0 for FL or Casp6-WT 16 h time point for LS and SS in bottom panel bar graphs. Data represent mean ± SD from three independent experiments. One-way ANOVA (p < 0.0001) followed by Bonferroni’s multiple comparison tests, ***p < 0.001, **p < 0.01, *p < 0.05 compares Casp6-WT with Casp6-R65W. (**g**) Western blot of full-length Lamin A/C, cleaved Lamin A/C and anti-β-actin from Casp6 KO total protein lysates cleaved by 50–400 nM recombinant active site titrated Casp6-WT, Casp6-R65W and Casp6-G66R. (**h**) Quantification of data in (g) expressed as a % of cleaved Lamin A/C fragments (normalized to β-actin) relative to the 400 nM concentration. Data represent mean ± SD from three independent experiments. One-way ANOVA (p < 0.0001) followed by Bonferroni’s multiple comparison tests, ***p < 0.001 and *p < 0.05 compares Casp6-R65W and Casp6-WT. Full-length images of gels and blots are provided in Supplementary Information.

To assess the proteolytic activity of the variants, recombinant active site titrated (Supplementary Fig. S1) enzymes were submitted to a fluorogenic assay with the Casp6-preferred Ac-VEID-AFC peptide substrate. Concentration-dependent cleavage of Ac-VEID-AFC by Casp6-R65W was achieved, but the Casp6-R65W activity was lower than that of Casp6-WT (Fig. 2a and b). No measurable VEIDase activity was detected with Casp6-G66R at enzyme concentrations from 10 nM to 500 nM (Fig. 2a), indicating that Casp6-G66R is catalytically inactive on Ac-VEID-AFC. Nevertheless, purified recombinant Casp6-G66R generated by co-expression of Casp6 G66R-LS and WT-SS in *E. coli* (Supplementary Fig. S2a), produced 2% VEIDase activity compared with Casp6-WT (Fig. 2c). These results show that R65W and G66R significantly suppress Casp6 activity on its preferred peptide substrate.

While its *k*_cat_ was unchanged, Casp6-R65W displayed approximately 2.5-fold lower *k*_cat_*/K*_M_ and a 2.6-fold higher *K*_M_ compared to Casp6-WT, indicating that it has a reduced binding affinity for Ac-VEID-AFC (Table 1). Thermal shift assays were performed in the absence of an active site ligand to assess the intrinsic stability of these variants. The results revealed that the intrinsic thermal stability of Casp6-R65W was essentially unchanged relative to Casp6-WT and the stability of Casp6-G66R was only decreased by 4 °C (Fig. 2d and e), suggesting that the changes in the activities of these variants was not due to global unfolding or misfolding of the proteins. However, in the presence of an excess amount of Ac-VEID-CHO substrate, a 7 °C stabilization of Casp6-WT was observed. The increase in stability upon incubation with Ac-VEID-CHO was lower for Casp6-R65W (4.6 °C) and absent for Casp6-G66R. This suggests that Casp6-R65W does bind substrate, albeit much less efficiently than Casp6-WT and that Casp6-G66R does not bind substrate to any appreciable extent. These observations are consistent with the observed catalytic parameters for Casp6-WT, -R65W and –G66R (Table 1).

**Table 1:** Comparative catalytic efficiencies.

	*K*_M_ (µM)	*k*_cat_ (s^−1^)	*k*_cat_/*K*_M_ (s^−1^ M^−1^)
Casp6-WT	39.1 ± 5.6	1.97 ± 0.05	51,090 ± 6,932
Casp6-R65W	102.0 ± 13.7^**^	2.03 ± 0.17	20,175 ± 3,437^**^
Casp6-G66R	ND	ND	ND

ND is not detectable, **p < 0.01 show catalytic constants of Casp6-R65W are significantly different from Casp6-WT (two-tailed, unpaired T-test). Data represent mean ± SD from three independent experiments.

The observed difference in Casp6-R65W self-processing and VEIDase activity compared to Casp6-WT prompted us to investigate Casp6-R65W and Casp6-WT time-dependent activities on catalytically inactive proCasp6-C163A (Fig. 2f top panel and Supplementary Fig. S2b). As expected, Casp6-WT almost completely removed the pro-domain (Pro) of proCasp6-C163A within two hours, resulting in a rapid disappearance of the full-length (FL) proCasp6-C163A. By contrast, Casp6-R65W generated pro-domain lacking form (ΔCasp6) of Casp6-C163A more slowly than Casp6-WT. These results indicate that the R65W substitution decreases Casp6 self-cleavage efficiency at the TETD_23_ site. As observed in self-processing, Casp6-R65W generated more LS cleaved at DEVD_179_ (Fig. 2f, middle panel) and the corresponding SS with linker (L-SS) (Fig. 2f, bottom panel) from proCasp6-C163A, than Casp6-WT. No significant difference was observed in the levels of SS generated by Casp6-R65W compared to Casp6-WT. These results indicate that Casp6-R65W processes the DVVD_179_ of proCasp6-C163A more efficiently than Casp6-WT. In order to test if R65W and G66R substitutions may alter Casp6 activity against natural protein substrates Lamin A/C, proteins extracted from Casp6 knock-out mice tissue were cleaved with increasing concentrations of Casp6-WT, Casp6-R65W and Casp6-G66R for two hours (Fig. 2g). Compared to Casp6-WT, Casp6-R65W generated 30–40% less cleaved Lamin A/C, whereas Casp6-G66R did not process Lamin A/C even at a concentration of 400 nM (Fig. 2h). Together, these results indicate that G66R completely abolishes and R65W significantly reduces Casp6 activity.

### Casp6-G66R is differentially processed by Casp6-WT and Casp3

**Figure 3:**
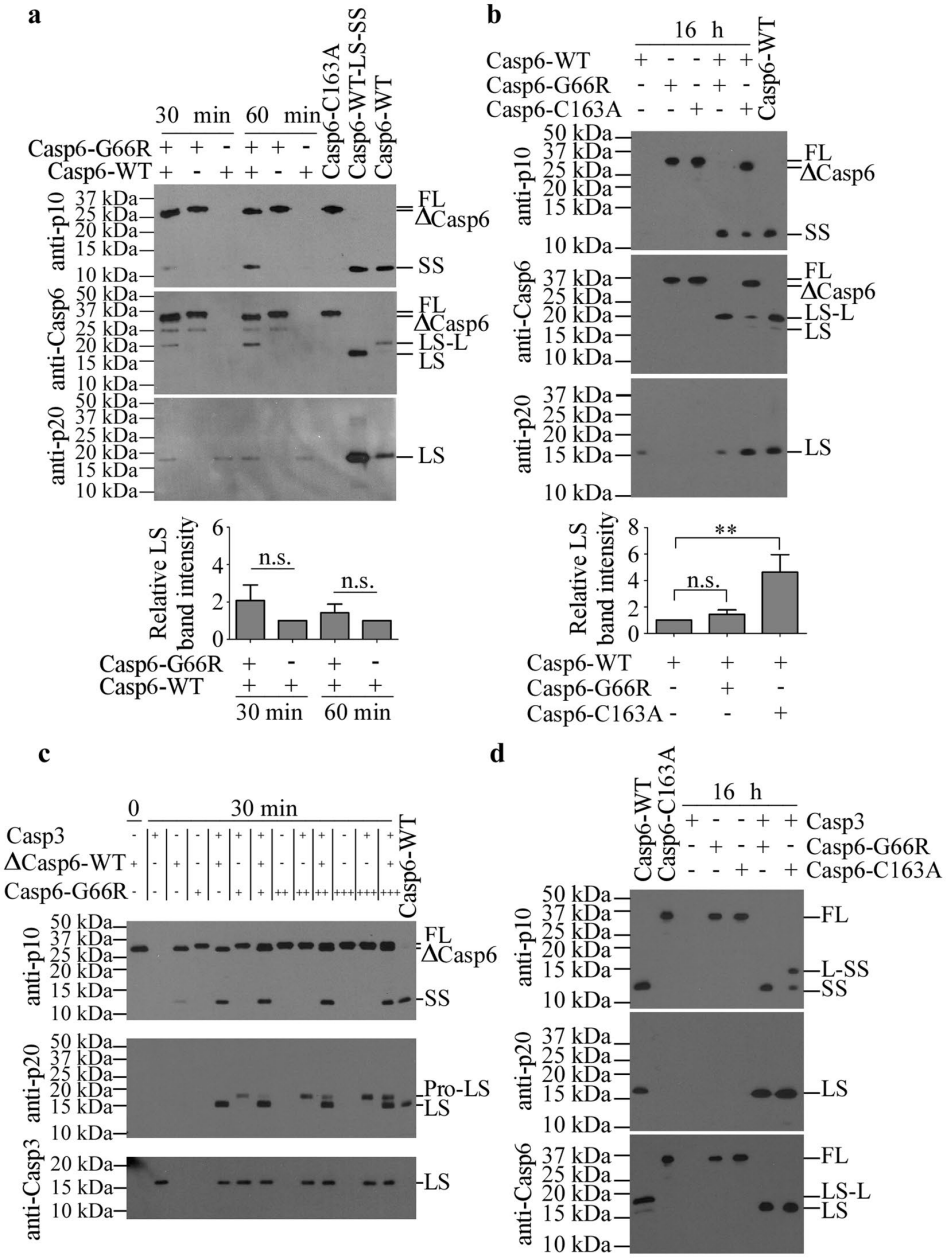
Exogenous caspase processing of Casp6-G66R. (**a**–**d**) Western blot detection of Casp6-G66R or Casp6-C163A cleaved by Casp6-WT (**a** and **b**) or Casp3 (**c** and **d**) for 30–60 min (**a** and **c**) or 16 h (**b** and **d**) with anti-p10, anti-Casp6 and neoepitope anti-p20 antiserum using unprocessed proCasp6-C163A and fully processed Casp6-WT as molecular weight marker controls. Quantification of LS generated in (**a** and **b**) and expressed as % of LS with only Casp6-WT (bottom panels). One-way ANOVA followed by Bonferroni’s (**a**) or Dunnett’s multiple comparison (**b**) against Casp6-WT. Not significant (n.s.), **p < 0.01. Data represent mean ± SD from three independent experiments. Full-length images of blots are provided in Supplementary Information.

To determine if Casp6-G66R is itself efficiently processed by exogenous active caspases, proCasp6-G66R was incubated with a low level of active Casp6-WT. Within 30–60 minutes, Casp6-WT cleaved Casp6-G66R at the TETD_23_ and TEVD_193_ sites, thereby generating ΔCasp6, LS-L and SS (Fig. 3a top panel). A small amount of LS from Casp6-WT, detected with the neoepitope anti-p20 antiserum, was not significantly increased in the presence of Casp6-G66R (Fig. 3a bottom panel). Processed Casp6-G66R remained inactive against the Ac-VEID-AFC peptide substrate (Supplementary Fig. S2c). Cleavage at DVVD_179_ was not observed even after a 16 h incubation period (Fig. 3b). In contrast, active Casp6-WT cleaved proCasp6-C163A at all three processing sites (Fig. 3b).

Furthermore, proCasp6-G66R was incubated with active Casp3 at 1:1, 2:1 and 3:1 ratios (Fig. 3c). After a 30 min incubation period, proCasp6-G66R was cleaved only at DVVD_179_ generating a LS slightly larger than expected but detected with the neoepitope antiserum suggesting that it had retained the pro-domain (Pro-LS). By contrast, Casp3 efficiently cleaved Casp6-WT at DVVD_179_ and TEVD_193_. The ability of Casp3 to cleave the pro-domain of Casp6-WT could not be determined in these experiments since the proCasp6-WT self-processed at the TETD_23_ site during purification and naturally generated the Casp6-WT lacking its pro-domain (∆Casp6-WT). After a 16 h incubation period, Casp3 completely cleaved proCasp6-G66R at all three processing sites (Fig. 3d). However, Casp3 cleaved proCasp6-C163A mostly at TETD_23_, DVVD_179_ and less efficiently at TEVD_193_ since L-SS was clearly present (Fig. 3d). Together, these results indicate that the G66R substitution influences the conformation of Casp6 resulting in altered processing by Casp3 or Casp6.

### Casp6-G66R acts as a dominant negative inhibitor of Casp6-WT

**Figure 4:**
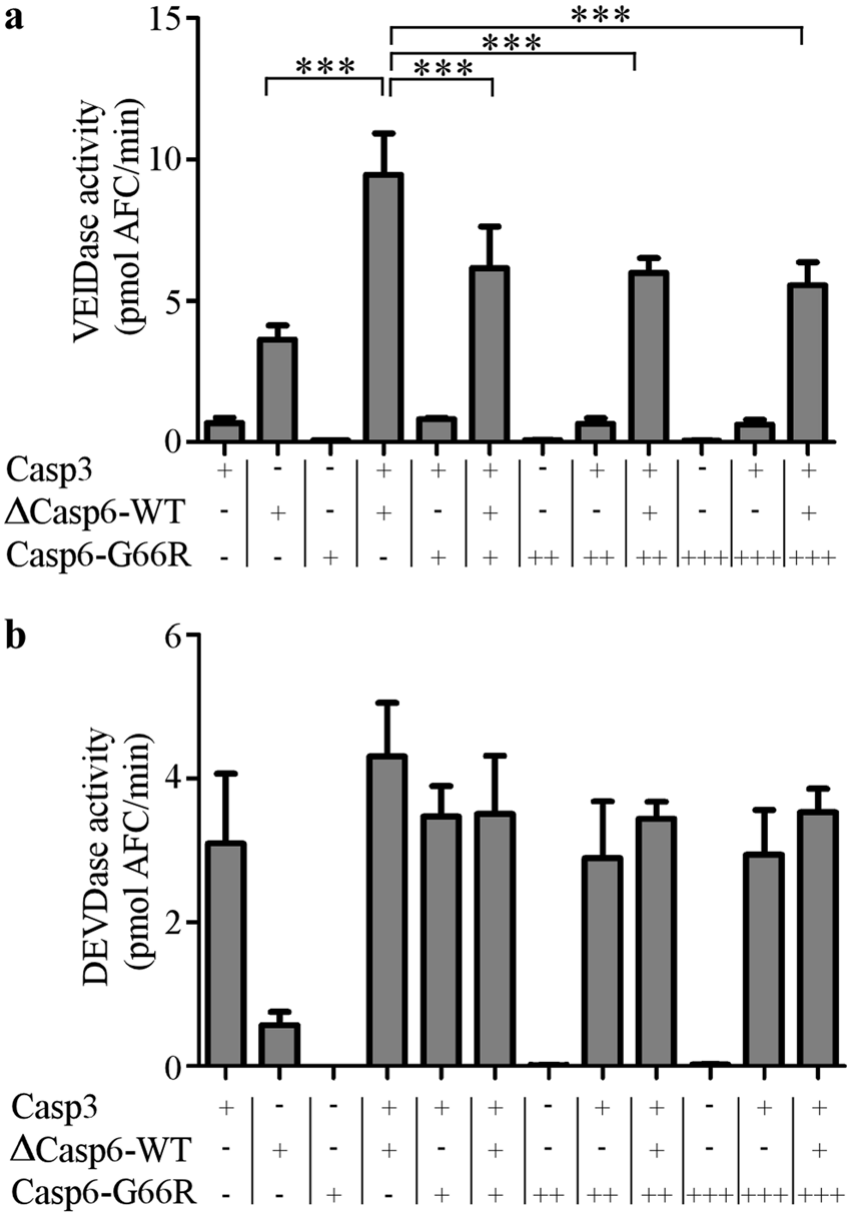
Casp6-G66R acts as a dominant negative inhibitor of Casp6-WT. VEIDase (**a**) or DEVDase (**b**) activity of Fig. 3c samples. One-way ANOVA (p < 0.0001) and Bonferroni’s multiple comparison tests, ***p < 0.001. Data represent mean ± SD from at least three independent experiments.

To test if Casp6-G66R can act as a dominant negative inhibitor of Casp6-WT activity, VEIDase activity was measured in a mixture of ΔCasp6-WT and Casp3-processed Casp6-G66R or ΔCasp6-WT (Figs 3c and 4a). Unprocessed Casp6-G66R did not inhibit Casp6-WT (30 nM) VEIDase activity (Supplementary Fig. S2d). As expected, active Casp3 showed a small amount of VEIDase activity whereas ∆Casp6-WT exhibited more VEIDase activity due to its ability to self-process. Casp3 cleavage of proCasp6-G66R did not increase VEIDase activity. However, Casp3 processing of ∆Casp6-WT increased VEIDase activity two-fold and this activity was significantly reduced by 30% with the addition of proCasp6-G66R, although not in a dose-dependent manner. Casp3 activity on its preferred Ac-DEVD-AFC substrate remained unaltered by the addition of proCasp6-G66R or ΔCasp6-WT (Fig. 4b). Addition of fully processed Casp6-G66R, generated by co-expression and purification of LS and SS from *E. coli*, to fully processed Casp6-WT, resulted in full Casp6-WT VEIDase activity (Supplementary Fig. S2e). These results support the previously proposed dominant negative inhibition mechanism for caspases where the LS of Casp6-WT and Casp6-G66R would associate to form an inactive enzyme.

### Casp6-R65W and Casp6-G66R are inactive in transfected mammalian cells

**Figure 5:**
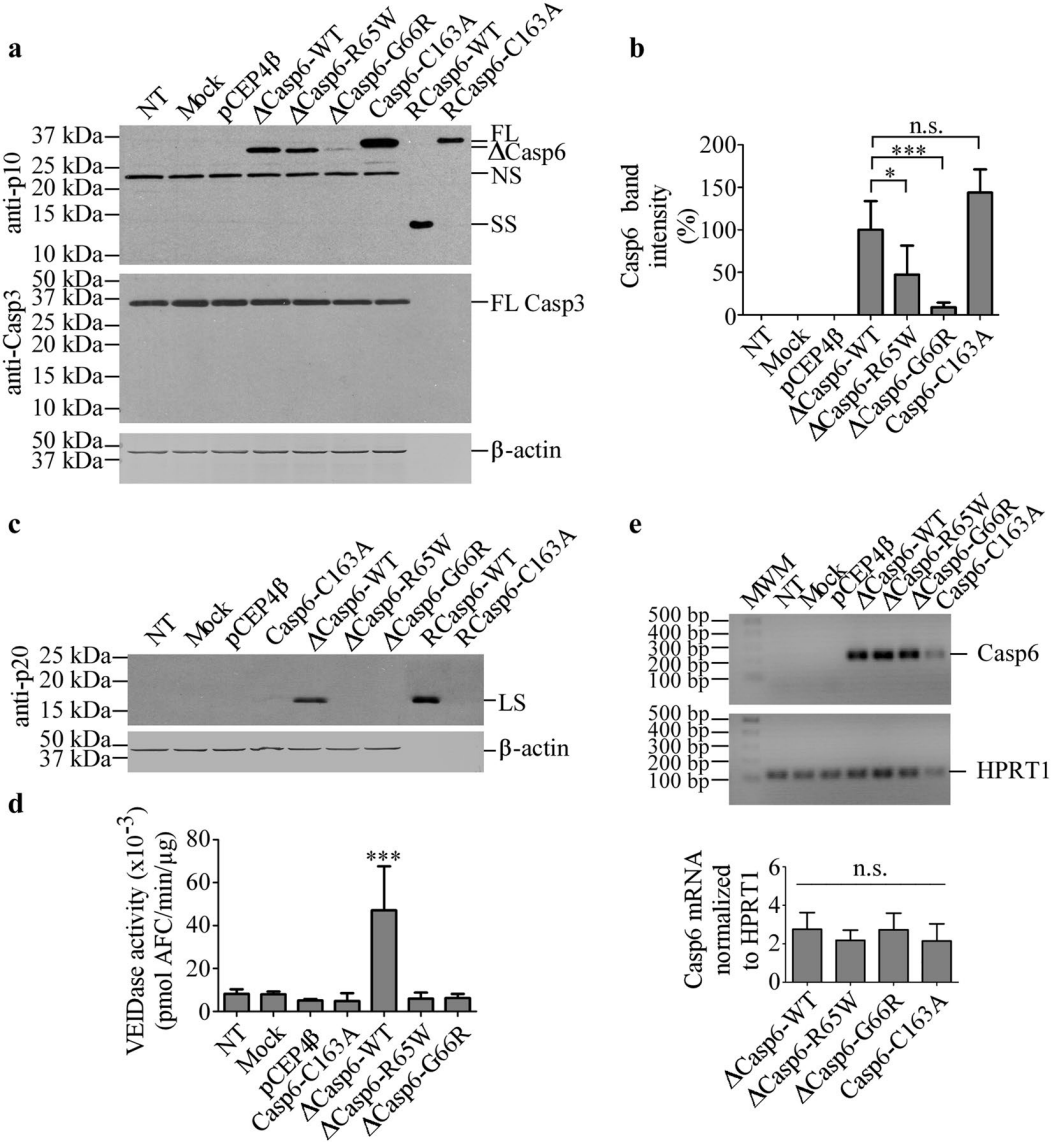
The activity of Casp6 expressed in mammalian HEK293T cells. (**a**) Western blot analyses of total protein extracts from untransfected (NT), mock-, pCEP4β-, or pCEP4β-Casp6-transfected HEK293T cells with anti-p10, anti-Casp3 and anti-β-actin antibodies. (**b**) Quantification of FL and ∆Casp6 protein band intensities in (a), normalized to β-actin and expressed as a percentage of ∆Casp6-WT. Data represent mean ± SD from three independent experiments. One-way ANOVA (p < 0.0001) and Dunnett’s multiple comparison analysis against ΔCasp6-WT levels ***p < 0.001 and *p < 0.05. (**c**) Western blot analyses of total protein extracts from untransfected (NT), mock-, pCEP4β-, or pCEP4β-Casp6-transfected HEK293T cells with anti-p20 antiserum and anti-β-actin antibody. (**d**) VEIDase activity of total protein extracts shown in (c). Data represent mean ± SD from three independent experiments. One-way ANOVA (p < 0.0001) followed by Bonferroni’s multiple comparison analysis, ***p < 0.001 (**e**) Ethidium bromide-stained (top panel) and Casp6 normalized to HPRT1 quantification (bottom panel) of RT-PCR human Casp6 and HPRT1 amplicons. Data represent mean ± SD from three independent experiments. One-way ANOVA followed by Dunnett’s multiple comparison test against ∆Casp6-WT. Full-length images of blots are provided in Supplementary Information.

To compare the activity of Casp6 variants with Casp6-WT in mammalian cells, pro-domain-lacking ∆Casp6-R65W, -G66R, or -WT were expressed in the human embryonic kidney 293 T cell (HEK293T) line. As expected, the ∆Casp6 proteins migrated slightly ahead of the FL proCasp6-C163A (Fig. 5a). Compared to ∆Casp6-WT transfected cells, steady state levels of ∆Casp6-R65W and ∆Casp6-G66R were significantly lower (Fig. 5b). Similarly, while Casp6-WT LS was detected, LS was almost undetectable from ∆Casp6-R65W and ∆Casp6-G66R (Fig. 5c). Consistently, Casp6 VEIDase activity was increased in proteins from ∆Casp6-WT-transfected cells, but not from ∆Casp6-G66R, ∆Casp6-R65W, or proCasp6-C163A-transfected cells (Fig. 5d). Expression of Casp6 in HEK293T cells did not result in Casp3 processing (Fig. 5a). Casp6 mRNA levels were equivalent in ∆Casp6-WT, ∆Casp6-R65W and ∆Casp6-G66R-transfected cells (Fig. 5e). These results suggest that the variant ∆Casp6-R65W and ∆Casp6-G66R proteins are either poorly translated or unstable in mammalian cells.

### Molecular dynamics simulations of Casp6-G66R and Casp6-R65W indicate perturbation of active site and substrate-recognition components

**Figure 6:**
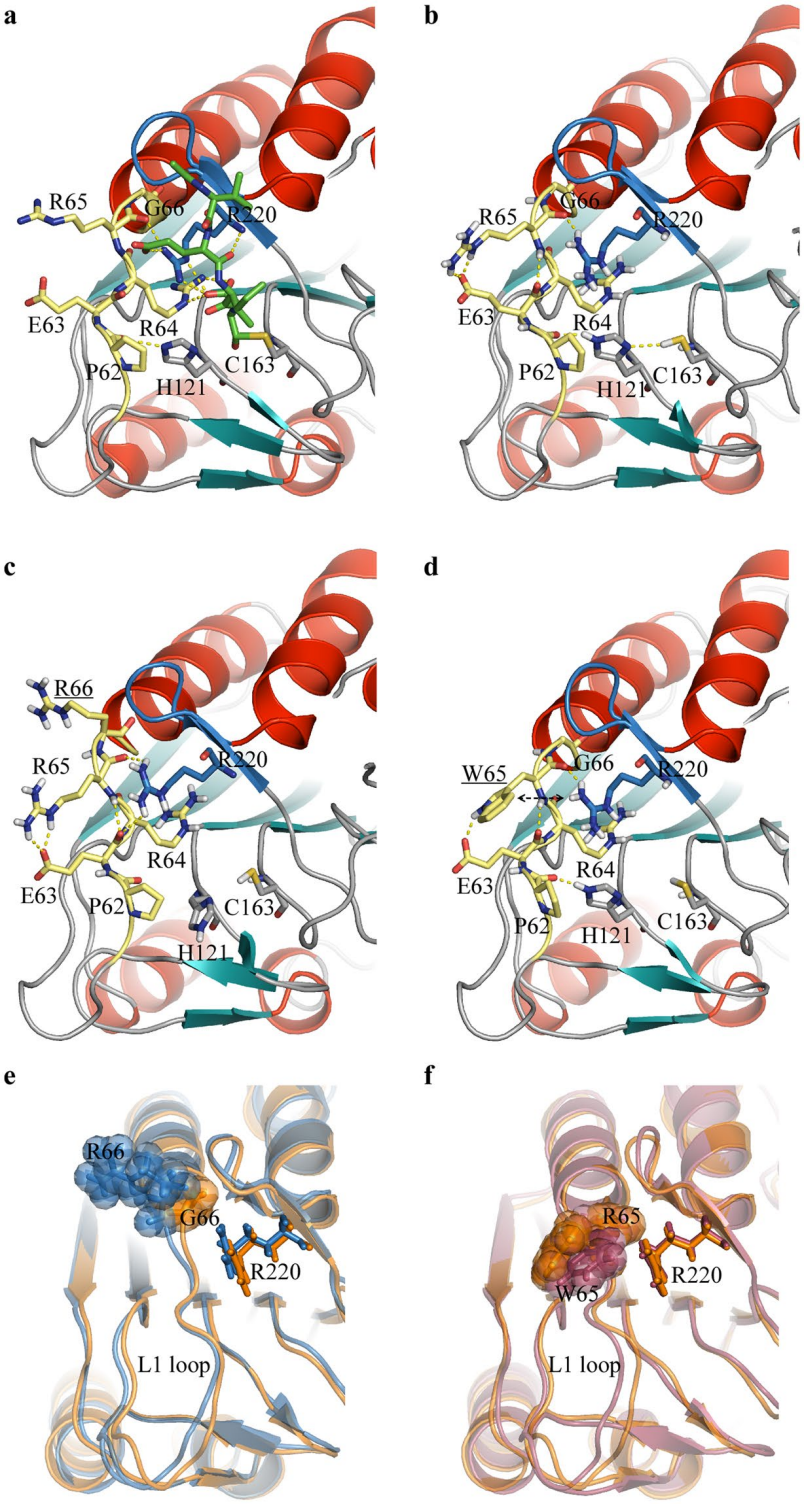
Structural features of Casp6 variants. Structural features of the active site in the catalysis-enabling form of ligand-bound mature Casp6 (pdb: 3OD5; **a**) and the corresponding representative conformational solutions displayed by Casp6-WT (**b**), Casp6-G66R (**c**) and Casp6-R65W (**d**), as predicted by molecular dynamics simulations. The backbone of the enzyme is rendered as a cartoon style and coloured by secondary structure (helices in red, strands in cyan and unstructured regions in grey). The covalently-linked Ac-VEID-CHO substrate mimetic in the (**a**) panel is shown with green carbons. Residues of importance for catalysis and affected by mutations are shown explicitly and labelled. Altered amino acids are underlined in panels c and d. Hydrogen bonds are indicated by dashed yellow lines, the cation-π contact (in d) is indicated by a dashed double-headed arrow, 61–66 region of L1 loop is shown in yellow and R220 hairpin is depicted in blue. (**e**) Superimposition of Casp6-WT (orange) with Casp6-G66R (blue) and (**f**) Casp6-R65W (magenta). Shown structures were predicted by molecular dynamics simulations. Amino acid residues at positions 65 and 66 are depicted in sticks with transparent spheres and R220 in R220-hairpin is shown in sticks.

Ligand-free models of mature Casp6-WT^6^, Casp6-R65W and Casp6-G66R were subjected to all-atom explicit-solvent molecular dynamics (MD) simulations to reveal structural alterations that may account for observed differences in stabilities and kinetic parameters. Alignment-based comparison of Casp6-WT original crystal structure (Fig. 6a) and MD-optimized Casp6-WT (Fig. 6b) and Casp6-G66R (Fig. 6c and e) models revealed that the backbone position of 61–66 fragment of the L1 loop deviates in Casp6-G66R from the position in Casp6-WT resulting in the perturbation of hydrogen-bonding between ε-N of catalytic His121 and carbonyl of the peptide bond between Pro62 and Glu63. Another notable topological difference is a significant deviation in the Arg220 hairpin position (Fig. 6e). Unlike G66 in Casp6-WT, the R66 in Casp6-G66R shifts away from the Arg220 hairpin since the bulky R66 points away towards the solvent to avoid steric hindrance with the Arg220 hairpin (Fig. 6e). In a Casp6-R65W model (Fig. 6d and f), a Trp65 indole is engaged in a cation-π interaction with Arg220, which is involved in the recognition of the P3 glutamate of the substrate (Fig. 6a) and, therefore, Arg220 is made less available for this important ionic contact. These outcomes could translate into both the increases in the corresponding *K*_M_ values and stability losses observed in the ligand-bound states of the Casp6 variants.

## Discussion

This work shows that studying natural Casp6 variants is a promising approach to uncover novel areas important for enzyme’s activity. Characterization of Casp6-R65W and Casp6-G66R reveals that R65W and G66R substitutions exhibit a destabilizing effect on the catalysis-enabling conformation of Casp6 and highlights the importance of previously uncharacterized R65-G66 region of L1 active site loop for Casp6 activity and substrate recognition.

Casp6 exists in a dynamic equilibrium between an inactive helix and substrate-binding induced active strand conformations^45^. During the transition from helix to strand forms, a region of 60’s helix comprising R65-G66 unfolds into L1 loop extension which together with four additional loops flank the Casp6 active site^5^. The replacements of helix-breaking Gly66 by helix-indifferent Arg and helix-indifferent Arg65 by helix-favouring Trp^46^ are both expected to stabilize the inactive conformation of Casp6. The strict conservation of G66 and the activity-abolishing effect of G66R substitution suggest that G66 may be crucial for providing L1 loop flexibility in the active sites of caspases. L1 loop conformation and mobility are known to control caspase activity. Mutations affecting Casp3 L1 loop dynamics reduce Casp3 activity^47^. Furthermore, calbindin-D28K anti-apoptotic protein inhibits Casp3 by locking the L1 loop in the position that occludes the Casp3 active site^48^. Similar restrained L1 loop position is observed in the inactive proCasp8 zymogen^49^. The G66R-induced pro-helical strain causes a slight repositioning of the 61–66 fragment of the L1 loop in Casp6-G66R, resulting in the loss of H-bond between the catalytic His121 and the carbonyl of the Pro62–Glu63 peptide bond. This contact, observed in crystal structures of both mature and proCasp6, has been implicated in serving a unique role of the third component in the catalytic triad-like arrangements of other caspases^36,50^. Importantly, this role is yet to be experimentally verified due to the participation of the backbone and this report provides the first instance of experimental evidence for supporting the hypothesis developed on the basis of static structure evaluation.

Our results suggest that R65W and G66R substitutions have a profound impact on Casp6 substrate recognition and strongly support the role of R65 in substrate binding. The R65W substitution significantly reduces Casp6 activity and affinity for VEID site, as observed with the Ac-VEID-AFC and Lamin A/C substrates. Human caspases prefer the glutamic acid at the P3 position of a peptide substrate^51^, since the strictly conserved R220 in the L3 loop binds the P3 side chain at the active site of Casp6^6^ and most other caspases^29–33^. Interestingly, Casp6’s L1 loop R65 can also mediate a water interaction with the P3 glutamate side chain^52^ like the R258 in the L1 loop of Casp8^32^. However, this hypothesis remains tentative in the absence of consensus for Chain A or B of the VEID-bound Casp6 structure. Among the three Casp6 processing sites, TETD_23_, TEVD_193_ and DVVD_179_, Casp6-WT intermolecularly cleaves the DVVD_179_ site with the least efficiency because the P3 is occupied by valine and not the preferred glutamate. Yet, Casp6-R65W self-processes at DVVD_179_ and cleaves Casp6-C163A-DVVD_179_ more efficiently suggesting that R65W substitution improves recognition of DVVD_179_. The preference of Casp6-R65W for DVVD_179_ over TETD_23_ could be rationalized by the shift from favouring complementary charge–charge (R_65_–E_21_) contact in the wild-type enzyme to hydrophobic–hydrophobic (W_65_–V_177_) interaction in the mutant. In Casp6-R65W a relatively hydrophobic Trp replaces a charged Arg65 in a fully solvent-exposed position. A partial seclusion of the electron-rich indole could be accomplished via a cation-π contact with R220, responsible for the accommodation of both P1 and P3 substrate sites, which, when combined with the loss of R65, a secondary cationic contact for P3, offers a compelling account for the increased *K*_M_ value with the peptide and native substrates for the Casp6-R65W. The replacement of fully buried and compact Gly66 in Casp6-G66R with the sterically demanding and charged Arg66 results in the reorganization of the R220-containing hairpin loop, thus negatively affecting substrate binding in the Casp6-G66R active site. This MD simulation-derived conclusion is strongly supported by the thermal shift assay findings. Furthermore, the observation that Casp6-G66R intersubunit linker TEVD_193_ site is more accessible for intermolecular cleavage with active Casp6 or Casp3 compared to Casp6-C163A further suggests that G66R impairs linker binding at the active site of Casp6^6^.

Bioinformatics tools, like PolyPhen^53^ and SIFT^54^, predicting effects of non-synonymous SNPs on protein function based on the analysis of multiple protein sequence alignments and protein three dimensional structures, suggest that R65W and G66R substitutions are deleterious to Casp6 function. Our experimental results support these predictions and strongly encourage the use of available computational tools for the initial estimation of mutation effect on protein function.

Casp6 is becoming an attractive therapeutic target for AD. Our study demonstrates that rare human *CASP6* SNPs, encoding R65W and G66R substitutions in Casp6, suppress Casp6 activity and self-activation. Likewise, rare missense SNPs in human *CASP1* gene associated with autoimmune conditions^55^ cause a reduction or abrogate auto-processing and activity of Casp1 *in vitro* and in HEK293T^56^. In contrast to these Casp1 variants, rare Casp6 variants are not known to be associated with any disease. The fact that Casp6 variants with decreased or no activity are compatible with normal life suggests that Casp6 is not essential for healthy humans. Casp6 plays a role in axon pruning during development^17,18^ and B cell activation and differentiation into plasmas cells^25^. Nevertheless, Casp6 knock-out mice develop normally^24^ and cell death defects have not been reported for Casp6 mutants, supporting the idea that the role of Casp6 in apoptosis is either redundant or compensated in the Casp6 knock-out models^57^. In contrast to Casp6 physiological functions, increased active Casp6 in the human adult brains has been associated with age-dependent cognitive impairment and AD^11–15^. Casp6 causes axonal degeneration in NGF-deprived mouse sensory and wild type and mutant amyloid precursor protein-transfected human CNS neurons^16–19^. ProCasp6 is barely detectable in healthy foetal and adult brain^23^. Therefore, the absence of an association between these human *CASP6* genetic variants and disease suggest that selective downregulation of Casp6 activity would unlikely cause serious side effects in humans.

Presently, there are 111 human *CASP6* SNPs coding for missense Casp6 variants reported in the NCBI, 70% of which have been validated but have not yet been associated with severe clinical outcomes. Taking into consideration that active Casp6 is associated with AD and cognitive impairment, it is tempting to speculate that *CASP6* SNPs described in this study could be associated with decreased risk of age-related Casp6-mediated neurodegeneration. In addition to finding SNPs that enhance the risk of AD, next-generation sequencing has allowed identification of rare SNPs linked to reduced risk of AD. The rare SNP coding for the Ala673Thr variant of amyloid precursor protein protects against AD and cognitive decline in the elderly^58^. Future systematic functional characterization of existing Casp6 polymorphic variants in combination with phenotypic information from healthy and disease-affected human subjects would help to reveal any causative or protective effects of *CASP6* SNPs on age-dependent cognitive impairment and AD.

## Methods

### Identification of non-synonymous SNPs in the human population

*CASP6* gene rare non-synonymous SNPs were identified in dbSNP NCBI database (https://www.ncbi.nlm.nih.gov/projects/SNP/snp_ref.cgi?geneId = 839).

### DNA constructs

The pET23b(+)-Casp6-His plasmid (Addgene, #11823) encoding full-length human Casp6-WT-6xHis-tag-C-terminus was a gift from Dr. Guy Salvesen (Sanford Burnham Prebys Medical Discovery Institute, CA, USA). The pET23b-Casp6(WT)p20/p10-His *E. coli* codon optimized plasmid co-expressing Casp6-WT large and small subunits^59^, mammalian pCEP4β-Casp6(WT)p20p10-His plasmid encoding Casp6-WT without pro-domain^7^ and recombinant pET23b(+) plasmid encoding Casp6-C163A^7^ were previously described. R65W or G66R mutations were introduced into pET23b(+)-Casp6-His and pCEP4β-Casp6(WT)p20p10 using QuikChange Site-Directed Mutagenesis Kit (Stratagene, La Jolla, CA, USA) with the following primers and corresponding reverse sequence: R65W: 5′-CTGCCAGAAAGGTGGGGCACCTGCG-3′, G66R: 5′-CCAGAAAGGCGGCGCACCTGC GCAG-3′ and into pET23b-Casp6(WT)p20/p10-His using R65W: 5′-CTGCCGGAACGTTGGGGTACCTGCGCG-3′, G66R 5′-CCGGAACGTCGTCGTACCTGCG CGGAC-3′. Plasmid encoding *E. coli* codon optimized prodomain (1–23) lacking ΔN Casp6-G66R was generated by introducing Casp6 linker into pET23b-Casp6(WT)p20/p10-His with primers 5′-AACATTACCGAGGTGGATGCGGCGAGCGTTTACACCCTG-3′ and 5′-GGTGTCCAGCTT CTCGGTCTGGTTGTCCACCACATCCAG-3′ through PCR and blunt end ligation with T4 DNA ligase (Thermo Fisher Scientific, Waltham, MA, USA) followed by site-directed mutagenesis with 5′-CCGGAACGTCGTCGTACCTGCGCGGAC-3′ primer and corresponding reverse sequence. Plasmids were sequenced by the Sanger method (McGill University and Genome Quebec Innovation Center, Montreal, QC, CA).

### Protein expression and purification

Full-length Casp6 was expressed at 30 °C in *E. coli* BL21(DE3)pLysS (Promega, Fitchburg, WI, USA) and purified using Ni Sepharose Fast Flow 6 (GE Healthcare, Little Chalfont, UK)^59^ using Tris buffers pH 8.0. Eluted Casp6 was diluted fivefold with buffer A1 (20 mM Tris pH 8.5, 2 mM DTT, 5% glycerol), loaded on Macro Prep High Q resin (Bio-Rad Laboratories, Hercules, CA, USA), washed with 20 mM Tris pH 8.5, 2 mM DTT, 5% glycerol, 50 mM NaCl and eluted with 80-260 mM NaCl gradient in buffer A1. Casp6 large and small subunits were co-expressed at 23 °C in *E. coli* BL21(DE3)pLysS and purified as described using Tris buffers pH 8.5. Casp6-C163A was purified as in^59^ and Casp3 as in^7^. Caspase purity was assessed by SDS-PAGE and Coomassie blue staining. Protein concentration was measured using Quick Start Bradford 1× Dye Reagent (Bio-Rad Laboratories).

### Active site titration

Casp6 and Casp3 active site concentrations (Supplementary Fig. S1) were determined using irreversible inhibitor zVAD-FMK (N-benzyloxycarbonyl-Val-Ala-Asp-(O-methyl)-fluoromethylketone, MP Biomedicals, Santa Ana, CA, USA)^60^. Caspases (320–380 nM) were incubated (2 h, RT) in Stennicke’s buffer (SB: 20 mM piperazine-N,N′-bis-(2-ethanesulfonic acid pH 7.2, 100 mM NaCl, 10 mM DTT, 1 mM EDTA, 0.1% CHAPS, 10% sucrose)^61^ with 0–2.5 µM zVAD-FMK, followed by 23.5-fold (Casp6) or 10-fold (Casp3) dilution with SB. Aliquots (25 µl Casp6 or 5 µl Casp3) were transferred to black clear bottom 96-well microplates (Costar, Corning, NY, USA), 10 µM Casp6 substrate Ac-Val-Glu-Ile-Asp-7-Amino-4-trifluoromethylcoumarin (Ac-VEID-AFC) or Casp3 substrate Ac-Asp-Glu-Val-Asp-7-Amino-4-trifluoromethylcoumarin (Ac-DEVD-AFC) (Enzo Life Sciences, Farmingdale, NY, USA) was added and fluorescence (ex/em 380/505 nm) was measured for 20 min at 37 °C using Synergy H4 Hybrid instrument (Biotek, Winooski, VT, USA). Released AFC was calculated from 0–1250 pmol AFC (Enzo Life Sciences) standard curve. Activity (released pmol AFC/min) was calculated from the linear phase of the assay and plotted as a function of zVAD-FMK concentration; the intersection between the linear region of the curve and X-axis indicates active site concentrations.

### Western blot analyses

Proteins were separated on 15% polyacrylamide SDS gel and transferred to polyvinylidene fluoride membrane using Trans-Blot Turbo Transfer system (Bio-Rad Laboratories). Purified Casp6-WT, Casp6-R65W and Casp6-G66R (100 ng) were probed with rabbit polyclonal neoepitope antiserum 10630 (1:10,000) against ^174^PLDVVD^179^ of active human Casp6 p20 subunit^11^, mouse monoclonal anti-Casp6 IgG1 clone B93–4 (1:250) against ^271^GKKQVPCFASMLTKK^285^ of human Casp6 p10 subunit (BD Biosciences, San Jose, CA, USA) and mouse monoclonal anti-Casp6 IgG1 SC81653 (1:1000) against human proCasp6 24–293 amino acids (Santa Cruz Biotechnology, Dallas, TX, USA). Full-length Lamin A/C, Casp6-cleaved Lamin A/C and β-actin were detected with rabbit Lamin A/C antibody 2032 (1:1000, Cell Signaling Technology), rabbit cleaved Lamin A/C (small subunit) 2035 (1:1000, Cell Signaling Technology) and mouse monoclonal anti-β-actin AC-15 (1:5000, Sigma, St. Louis, MO, USA), respectively. Caspase-3 was detected with rabbit monoclonal (1:1000) antibody against full-length and active Casp3 large subunit cleaved at D175 (Cell Signaling Technology, Danvers, MA, USA). Antibodies were diluted in Tris-buffered saline containing 0.1% Tween-20 and 5% non-fat dry milk. Immunoreactive proteins were detected with HRP-conjugated secondary anti-mouse (1:5000, Jackson ImmunoResearch Laboratories, West Grove, PA, USA) and anti-rabbit (1:5000, Dako, Glostrup, Denmark) antibodies, followed by ECL detection (Amersham, GE Healthcare) on Carestream Biomax MR films (Kodak, Rochester, NY, USA), or with secondary anti-mouse (1:5000, Jackson ImmunoResearch Laboratories) antibody conjugated to alkaline phosphatase and nitro blue tetrazolium/5-bromo-4-chloro-3-indolyl-phosphate (Thermo Fisher Scientific). Western blot films were scanned with an HP scanner and densitometry was performed using ImageJ software (NIH, Bethesda, MD, USA) by measuring band intensity values above background.

### Caspase activity assays

Casp6 activity was measured in SB with Ac-VEID-AFC substrate^60^. Assays were performed with 40 µM Ac-VEID-AFC and 10-500 nM active site titrated Casp6. Initial velocities of Ac-VEID-AFC hydrolysis were measured with 20 nM Casp6 and 1-300 µM Ac-VEID-AFC as described above. Initial velocities v versus substrate concentration [S] were fit to a rectangular hyperbola using Michaelis-Menten equation v = (v_max_ × [S])/(*K*_M_ + [S]) in Prism (GraphPad) software and apparent kinetic parameters v_max_ (maximal velocity of reaction at saturating substrate concentrations), *K*_M_ (Michaelis-Menten constant) and *k*_cat_ (turnover number, *k*_cat_ = v_max_/[E], where [E] is the Casp6 active site concentration) were calculated.

### Evaluation of Casp6-G66R activity after processed with Casp6-WT and measurement of Casp6-WT activity in the presence of Casp6-G66R

Casp6-G66R (0.6 µg) was incubated with Casp6-WT (0.0125 µg) in SB at 37 °C for 30 or 60 min in 12.5 µl volume. Afterward, samples were diluted fivefold with SB and 5 µl aliquots were used in Casp6 activity assay with Ac-VEID-AFC, or analysed by western blot. To investigate Casp6-WT inhibition with Casp6-G66R, activity of 30 nM Casp6-WT or co-expressed and purified WT-LS and WT-SS was measured in SB with 0–100 nM of unprocessed Casp6-G66R or fully processed Casp6-G66R (generated by co-expression and purification of G66R-LS and WT-SS) in the presence of 40 µM VEID-AFC.

### Processing of Casp6-G66R and proCasp6-WT with Casp3

Unprocessed proCasp6-WT was expressed and purified as described^6^. ProCasp6-WT (0.02 mg/ml) was incubated (30 min, 37 °C) with 0.02 mg/ml, 0.04 mg/ml, or 0.06 mg/ml Casp6-G66R in SB with 0.24 mg/ml BSA and 2 ng/µl Casp3. Samples were used in activity assays with 40 µM Ac-VEID-AFC or Ac-DEVD-AFC and analysed by western blot.

### Overnight cleavage of Casp6-G66R and Casp6-C163A with Casp3 and Casp6-WT

Casp6-G66R or Casp6-C163A (0.04 mg/ml) were incubated with 3 ng/µl active site titrated Casp3 or Casp6-WT (Supplementary Fig. S1) in SB with 0.24 mg/ml BSA for 16 hours at 37 °C and analysed by western blot.

### Thermal shift assays

ΔN D179 CT^5^ version of Casp6-WT, co-expressed Casp6-R65W large and small subunits and prodomain lacking ΔN Casp6-G66R described above were purified^45^. Casp6 (10 μM) was incubated in 20 mM Tris, pH 8.5 with 5 mM DTT with or without 50 μM Ac-VEID-CHO active-site inhibitor (Enzo Life Sciences) and 5 × SYPRO^®^ Orange dye (Thermo Fisher Scientific) in a 60 μL reaction. Fluorescence (ex/em 490/575 nm) was measured in a 96-well plate using a CFX Connect Real-Time PCR instrument (BioRad). RFU recorded from 25 to 95 °C with 0.5 °C/3 second intervals were normalized to the highest observed intensity which was set to 1. The normalized fluorescence was fit to a Boltzmann sigmoidal curve using Prism (GraphPad) software. Melting temperature (T_m_) was found to be the temperature at the midpoint of the denaturation curve.

### Casp6 activity in transfected mammalian cells

HEK293T 300,000 cells/well were plated in DMEM (Gibco) with 10% FBS (Wisent Bioproducts, St-Bruno, QC, CA) and incubated in a humidified incubator (5% CO_2_, 37 °C, 24 h). Before transfection, the medium was exchanged with 300 μl Opti-MEM (Gibco). Cells were transfected with one µg of plasmid DNA using Lipofectamine^®^2000 (Invitrogen, Carlsbad, CA, USA). Medium was replaced with DMEM/10% FBS. Cells were harvested 24 h after transfection. For Casp6 activity assays, proteins were extracted in cell lysis buffer (50 mM HEPES, 0.1% CHAPS, 0.1 mM EDTA, 1 mM DTT) with protease inhibitors (0.5 μg/ml leupeptin, 1 μg/ml TLCK, 0.1 μg/ml pepstatin A, 38 μg/ml AEBSF) and protein concentration was determined using Bradford method. For western blot analysis, proteins were extracted in RIPA buffer (150 mM NaCl, 0.5% (w/v) Na-deoxycholate, 0.1% (w/v) SDS, 1% Nonidet P-40, 100 mM Tris pH 8.0) with protease inhibitors.

### Casp6 cleavage of natural protein substrates

To assess Lamin A/C cleavage by Casp6, proteins from colon tissue of Casp6 Knock-Out (KO) mice^62^ were extracted in RIPA buffer with protease inhibitors (38 µg/ml AEBSF, 0.5 µg/ml leupeptin, 0.1 µg/ml pepstatin A). Tissue was lysed by sonication (50% duty, output 3, 30 s, 4 °C) with a Branson Sonifier 450 (Branson Ultrasonics, Danbury, CT, USA). The lysate was clarified by centrifugation (16,000 × *g*, 10 min, 4 °C) and protein concentration was measured using Bradford method. Two micrograms of proteins were incubated with 400 nM Casp6 in SB at 37 °C for 2.25 h. Samples were separated on 15% SDS-PAGE gels and analysed by western blot. To evaluate Casp6-C163A cleavage by Casp6-WT and Casp6-R65W, 0.5 µg/µl Casp6-C163A was incubated in SB with 0.5 ng/µl active-site titrated Casp6 at 37 °C for 1–16 hours. Samples were separated on 15% SDS-PAGE gels and stained with Coomassie blue or analysed by western blot.

### RT-PCR analysis

Total RNA was extracted 24 h post-transfection from HEK293T cells using TRIzol (Invitrogen). cDNA was prepared using avian myeloblastosis virus reverse transcriptase (Roche Applied Science) and used as a template to amplify *CASP6* with primers 5′-CGATGTGCCAGTCATTCCTT-3′ and 5′-CTCTAAGGAGGAGCCATATTTTC-3′ and *HPRT1* with primers 5′-CCTGGCGTGGTGATTAGTGAT-3′ and 5′-AGACGTTCAGTCCTGTCCATAA-3′. PCR products were analysed on 1.5% agarose gel electrophoresis and visualized by ethidium bromide staining.

### Bioinformatic analyses

Multiple protein sequence alignment was performed with Clustal Omega tool (http://www.ebi.ac.uk/Tools/msa/clustalo/) using the following Uniprot accession numbers: P29466, Q92851, P42575, P42574, P49662, P51878, P55212, Q14790, P55211, P55210; P55212, K7B3Z2, Q3T0P5, S9XS51, A0A0B8S0B0, O35397, O08738, L5K505, S7N3D7, A0A0Q4ALF8, O93415, A0A0Q3PLE0, Q9IB66, V9L2A1, Q9I8S9, Q52KK6, G0XQH6, Q9NHF9, K1R808. Illustrations of Casp6 structures were generated with PyMOL Molecular Graphics System (Version 1.8, Schrödinger, LLC).

### Molecular dynamics simulations

The mature Casp6 3D model was generated from Ac-VEID-CHO-bound Casp6 crystal structure (pdb: 3OD5)^6^. Upon removal of the ligand and replacement of the mutated residues, the all-atom structures of Casp6-WT, Casp6-G66R, Casp6-R65W with optimized protonation, tautomeric (Epik, Schrödinger, LLC) and locally minimized conformational (Force field: OPLS3; Prime, Schrödinger, LLC) states were generated using Protein Preparation Wizard (Maestro, Schrödinger, LLC). Resulting structures were refined and permitted to relax via a molecular dynamics simulation sequence within Desmond (version4.0, D. E. Shaw Research & Schrödinger, LLC). Thus, NVT ensembles for each Casp6 form were built by neutralization of the charge by Cl^−^ ions (4, 6 and 2 for Casp6-WT, Casp6-G66R and Casp6-R65W, respectively) and addition of further 38 Na^+^ and Cl^−^ ions to simulate 150 mM concentration in an explicit SPC solvent model (13,502 water molecules), using an orthorhombic simulation box (10 Å buffer) with periodic boundary conditions. Resulting ensembles were subjected to an unconstrained molecular dynamics simulation at 300 K and 1 atm. The structure from the final frames of the 1.2-ns simulation were re-minimized (OPLS3) and aligned using all residues as references within Protein Structure Alignment panel of Maestro.

### Statistical analyses

Statistical evaluations were performed with one-way ANOVA or T-test, as indicated in Figure legends, using Prism (GraphPad) software. All experiments were repeated a minimum of three times.

### Data availability

All generated or analysed study data are included in this published article and its Supplementary Information files.

## Electronic supplementary material

Supplementary information file
