# Supplementary material for: Rare human Caspase-6-R65W and Caspase-6-G66R variants identify a novel regulatory region of Caspase-6 activity
Source: Sci Rep. 2018 Mar 13;8:4428. doi: 10.1038/s41598-018-22283-z (PMC5849602; doi:10.1038/s41598-018-22283-z)
Supplement: Supplementary file 1 — Supplementary information file [file 41598_2018_22283_MOESM1_ESM.pdf]

Rare human Caspase-6-R65W and Caspase-6-G66R variants identify a novel regulatory region of Caspase-6 activity.

Agne Tubeleviciute-Aydin[1,2], Libin Zhou[1,3], Gyanesh Sharma[1,2], Ishankumar V. Soni[4], Sergey N. Savinov[5], Jeanne A. Hardy[4] and Andrea C. LeBlanc*[1,2,3]

[1] Bloomfield Center for Research in Aging, Lady Davis Institute for Medical Research, Jewish General Hospital, 3755 Ch. Cote Ste-Catherine, Montreal, QC, Canada H3T 1E2

[2] Department of Neurology and Neurosurgery, McGill University, 3775 University St., Montreal, QC, Canada H3A 2B4

[3] Department of Anatomy and Cell Biology, McGill University, 3640 University St., Montreal, QC, Canada H3A 0C7

[4] Department of Chemistry, University of Massachusetts Amherst, 710 N Pleasant St., Amherst, MA USA 01003

[5] Department of Biochemistry and Molecular Biology, University of Massachusetts Amherst, 240 Thatcher Way, Amherst, MA USA 01003

***Corresponding author:** Andrea LeBlanc, Ph.D., Bloomfield Center for Research in Aging, Lady Davis Institute for Medical Research, Sir Mortimer B Davis Jewish General Hospital, 3755 Ch. Côte-Ste-Catherine, Montréal, QC, Canada H3T 1E2. Tel.: +1 (514) 340 28222 ext 24976. Fax.: +1 (514) 340 8295. E-mail address: andrea.leblanc@mcgill.ca

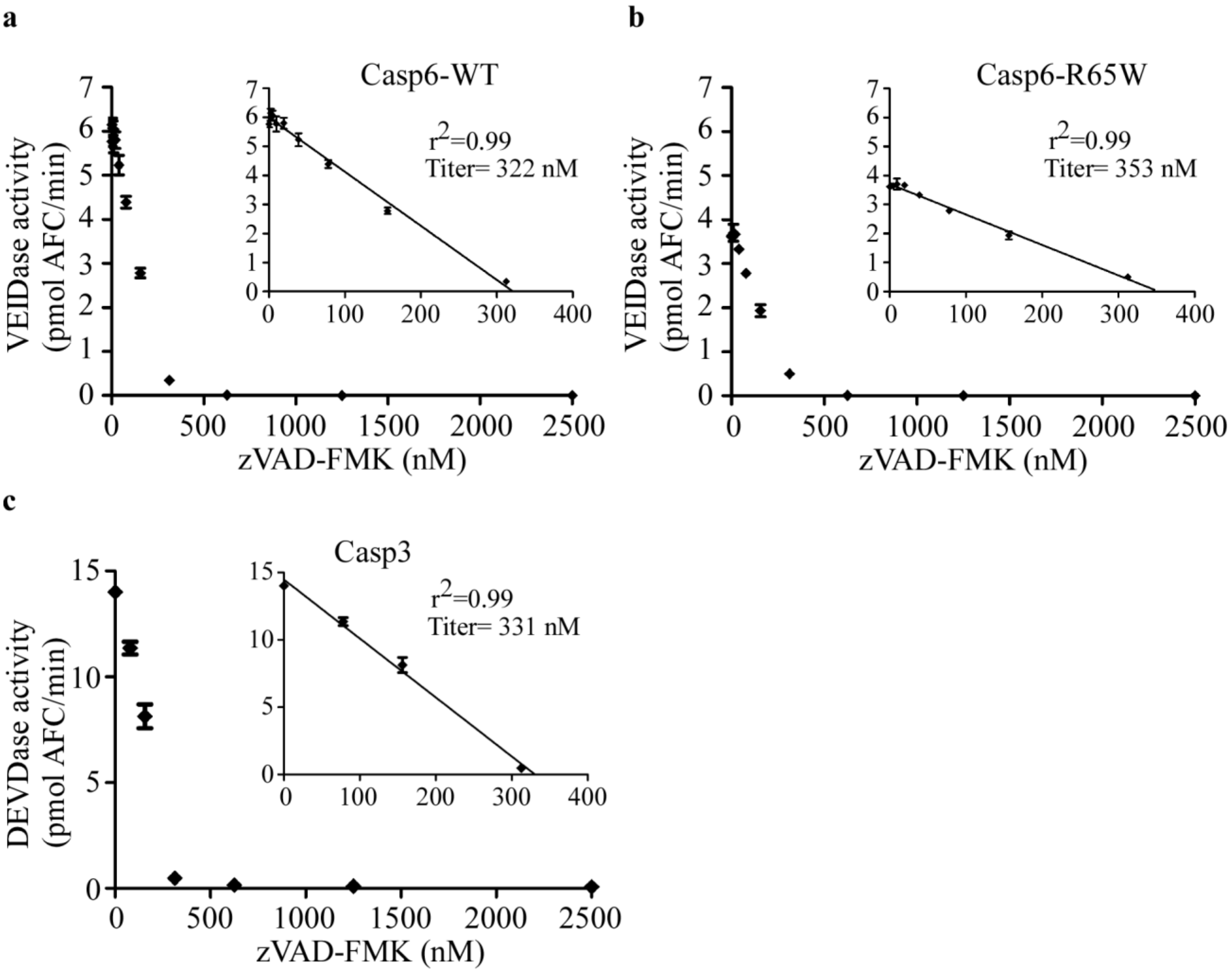

Figure S1. **Active site titration of purified recombinant caspases.** Active site titration of recombinant Casp6-WT (327 nM) (**a**), Casp6-R65W (381 nM) (**b**) and Casp3 (320 nM) (**c**) with irreversible inhibitor zVAD-FMK. The inlets of the graphs show linear regression of data points corresponding to 0-312.5 nM of zVAD-FMK, the x-axis intercept (titer) is equal to the active caspase concentration in the titration assay. Obtained titer values indicate that purified recombinant caspases are more than 92% active. The data points represent mean ± standard deviation (SD) from three independent experiments.

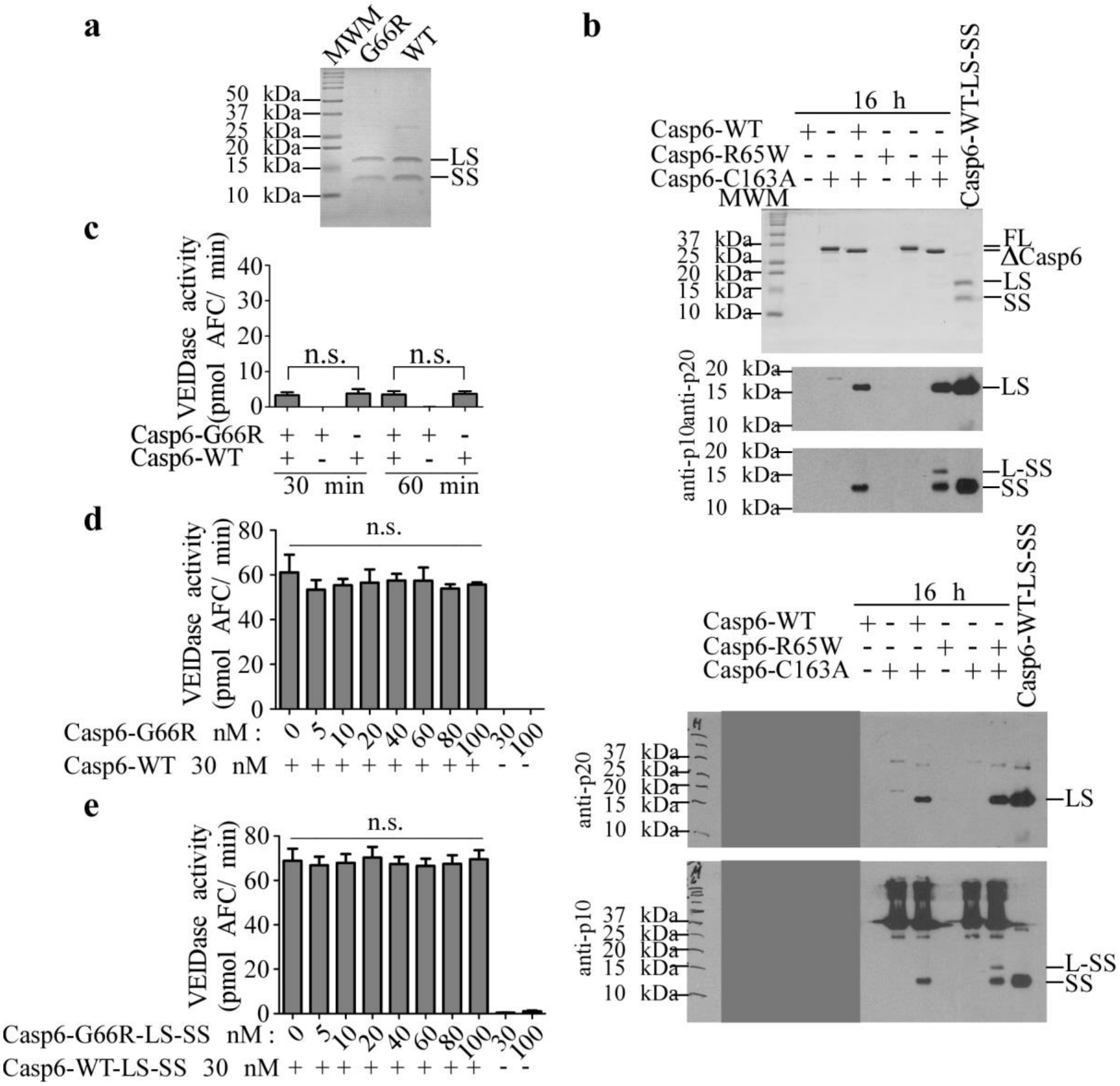

Figure S2. (**a**) Coomassie stained gel of co-expressed LS and SS from Casp6-G66R and Casp6-WT. (**b**) Coomassie stained gel (top panel), and western blot analyses with neoepitope anti-p20 antiserum (middle panel) and anti-p10 antibody (bottom panel). Full-length blots of cropped blots are shown in the bottom panel. (**c**) VEIDase activity generated by Casp6-WT processing of Casp6-G66R of samples in Fig. 3a. One-way ANOVA ($p<0.0001$) followed by Bonferroni's multiple comparison tests. Data represent mean ± SD from three independent experiments. VEIDase activity of Casp6-WT (**d**) and fully-processed Casp6-WT-LS-SS (**e**) in the presence of various concentrations of unprocessed Casp6-G66R (**d**) or fully-processed Casp6-G66R-LS-SS (**e**). One-way ANOVA ($p<0.0001$) followed by Dunnett's multiple comparison tests, n.s, not significant. Data represent mean ± SD from three independent experiments.

Figure 1f

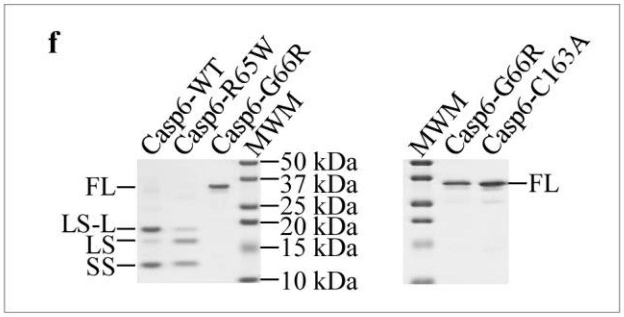

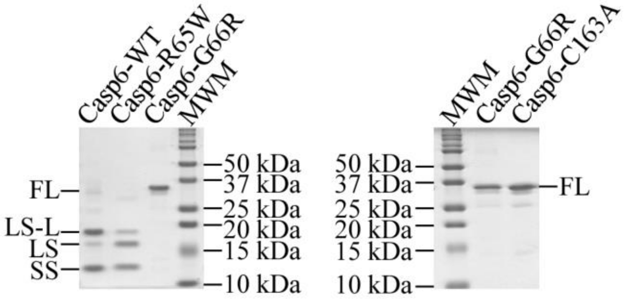

Figure 1g

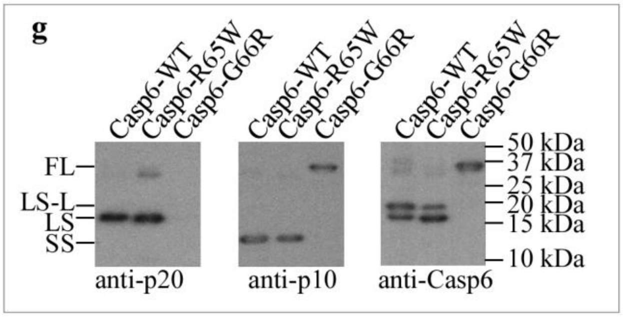

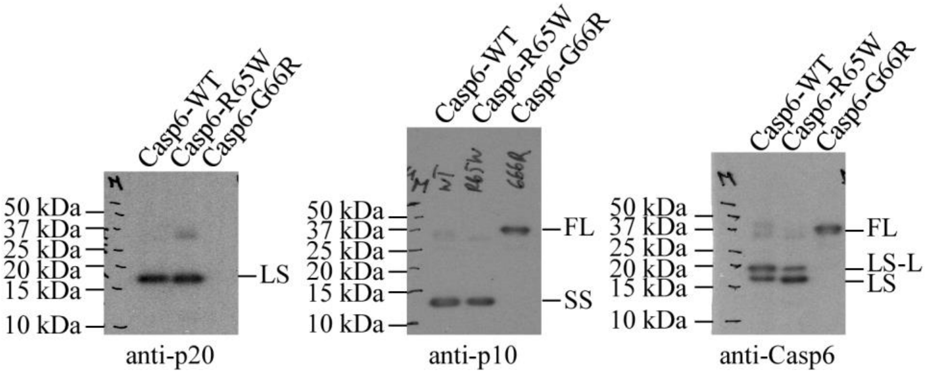

Figure S3. Full-length gels and blots of figures 1f and 1g.

Figure 2f

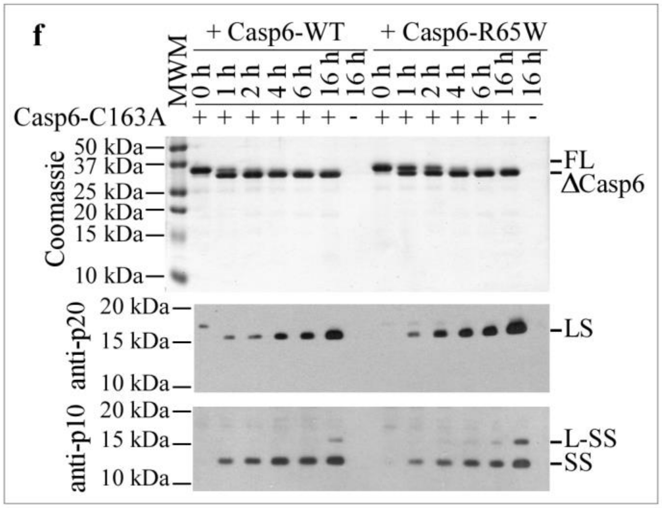

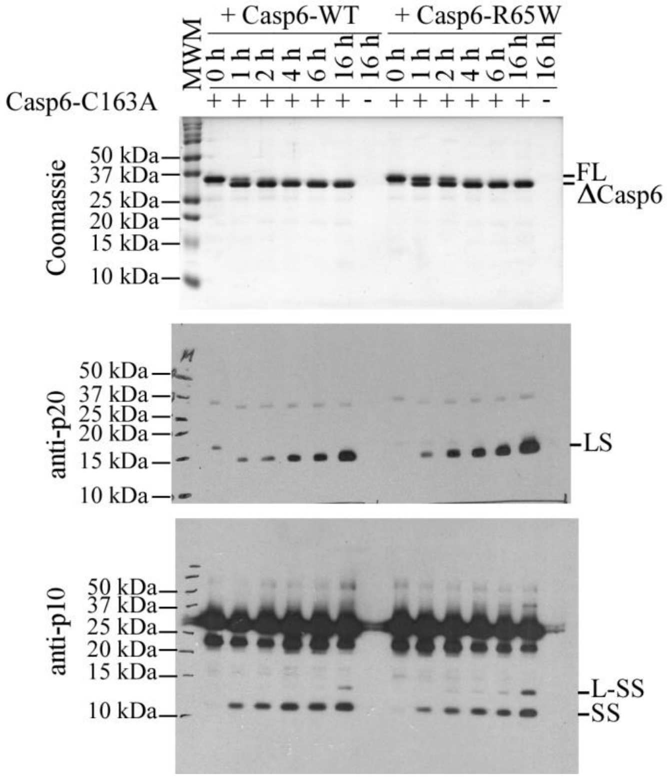

Figure S4. Full-length gels and blots of figure 2f.

Figure 2g

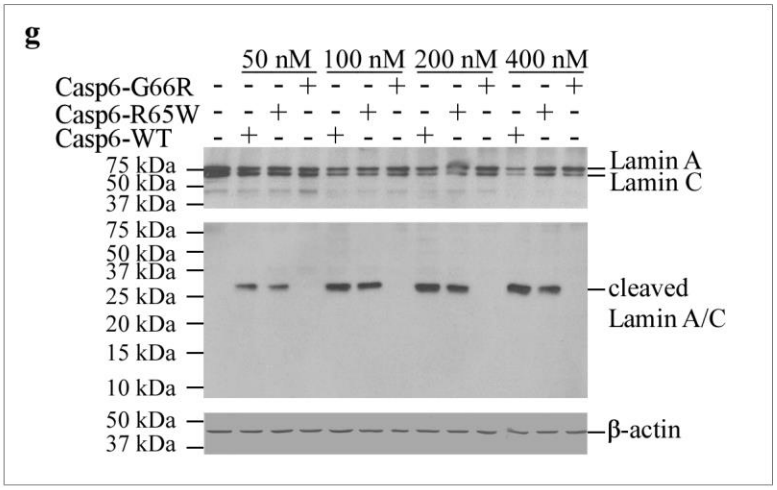

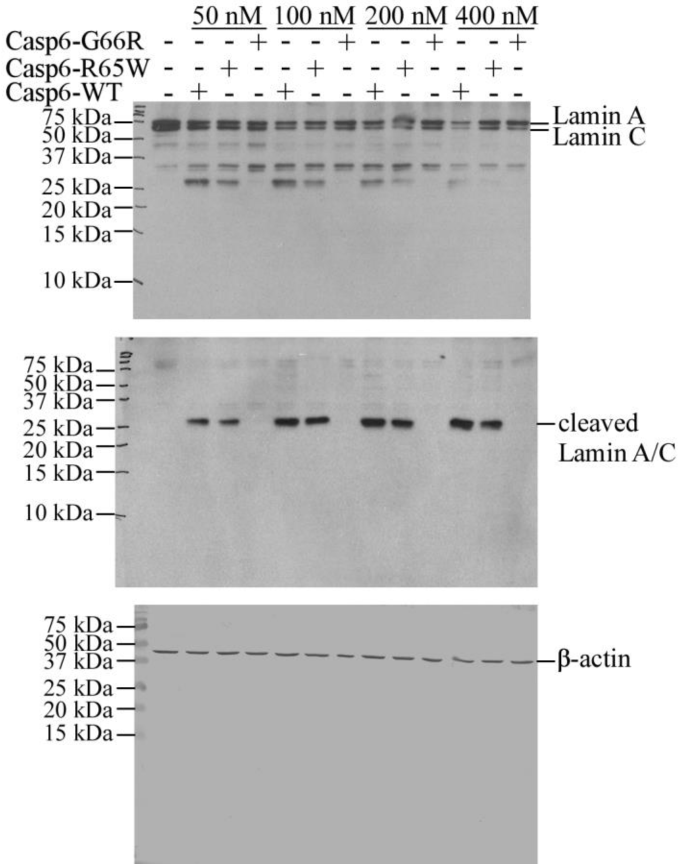

Figure S5. Full-length blots of figure 2g.

Figure 3a

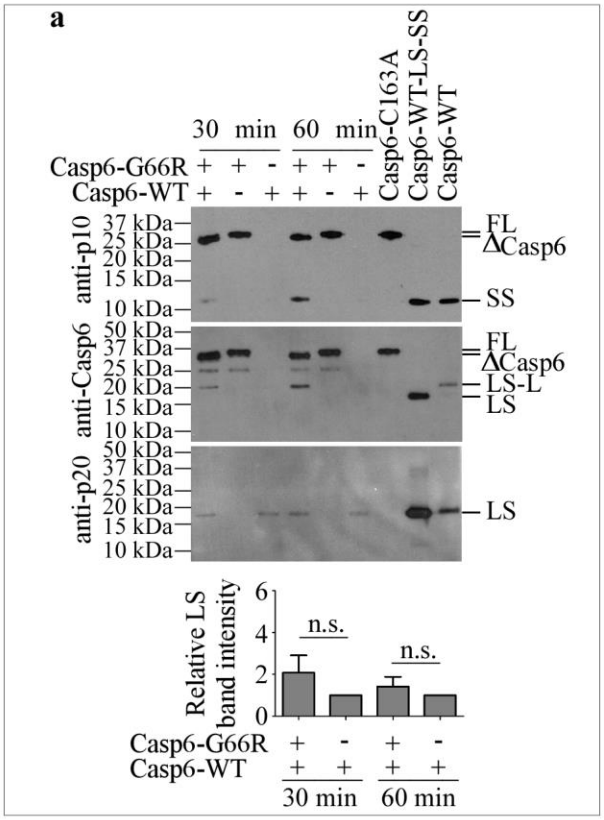

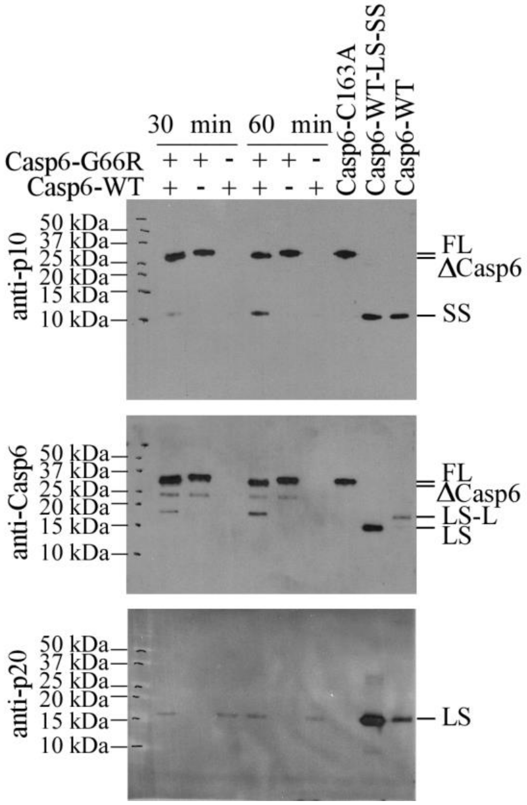

Figure S6. Full-length blots of figure 3a.

Figure 3b

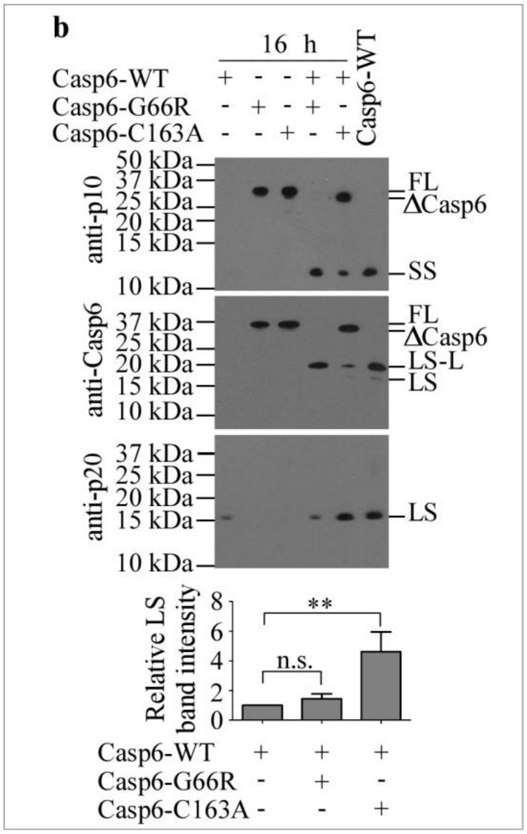

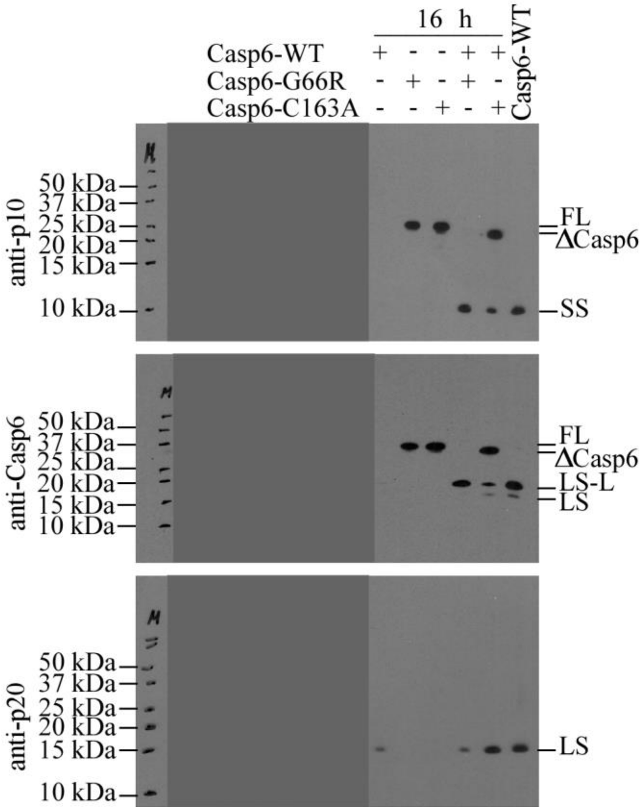

Figure S7. Full-length blots of figure 3b.

Figure 3c

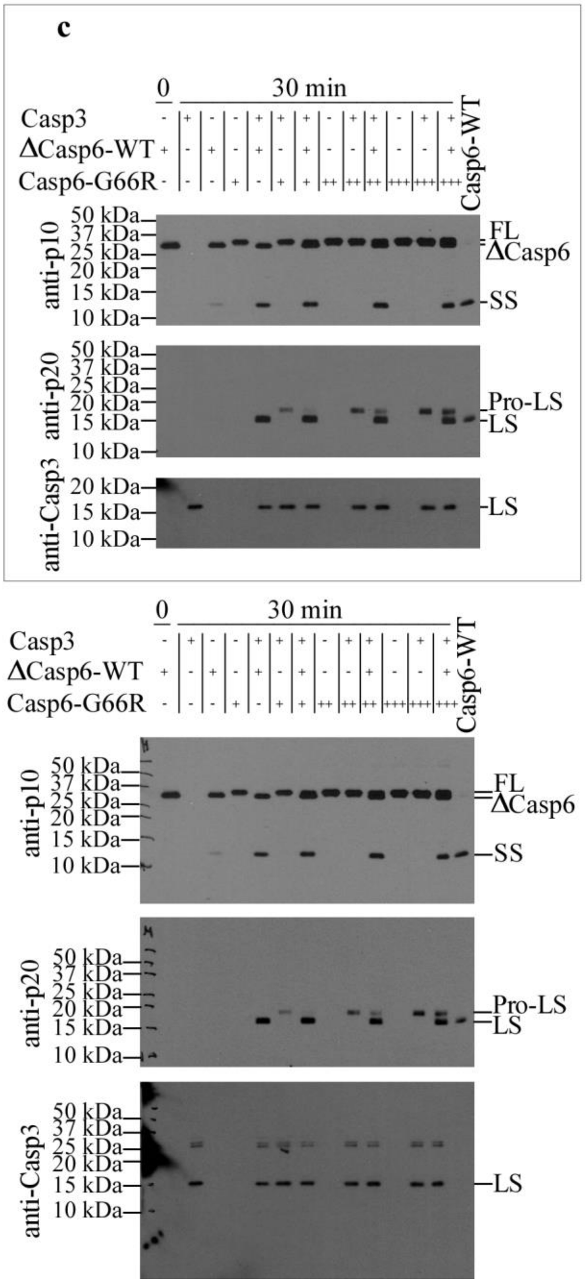

Figure S8. Full-length blots of figure 3c.

Figure 3d

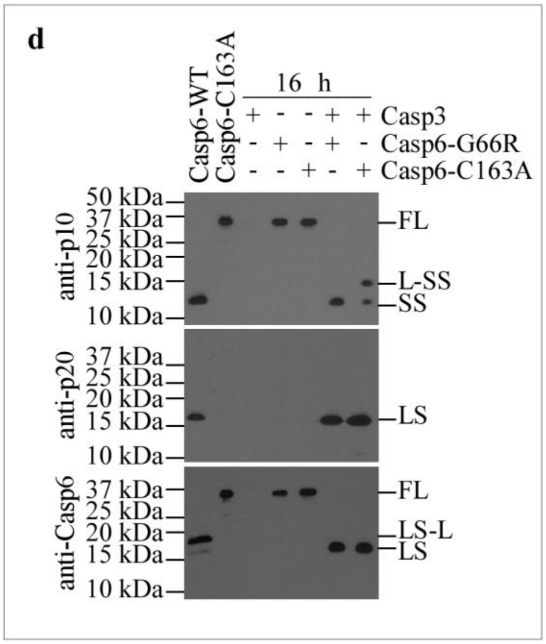

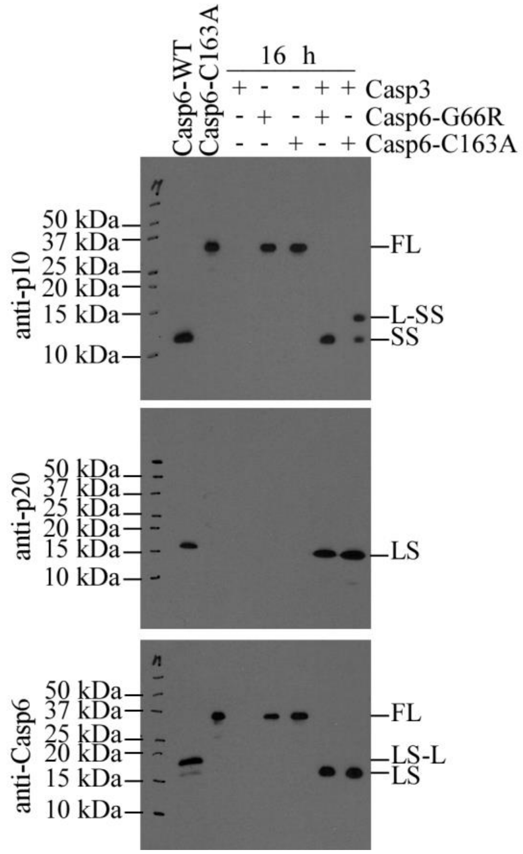

Figure S9. Full-length blots of figure 3d.

Figure 5a

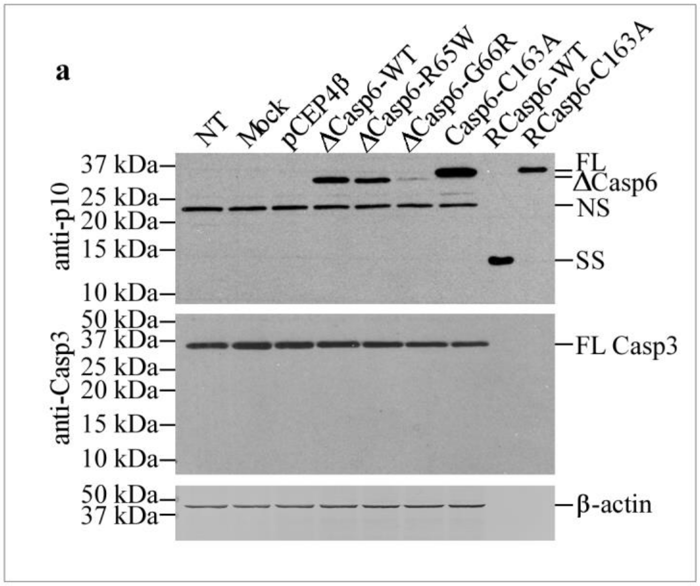

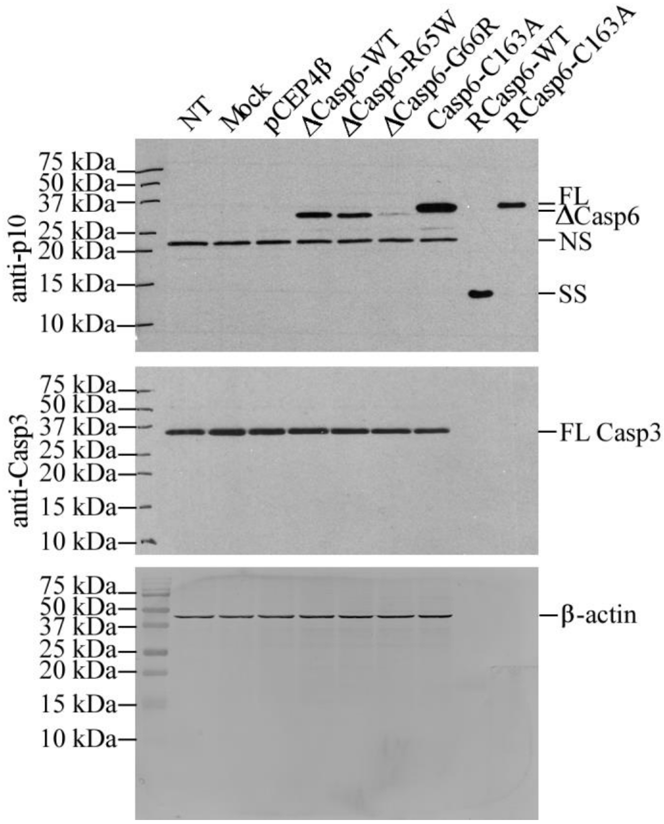

Figure S10. Full-length blots of figure 5a.

Figure 5c

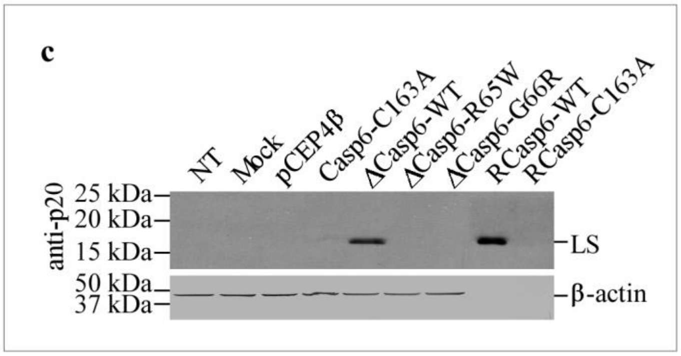

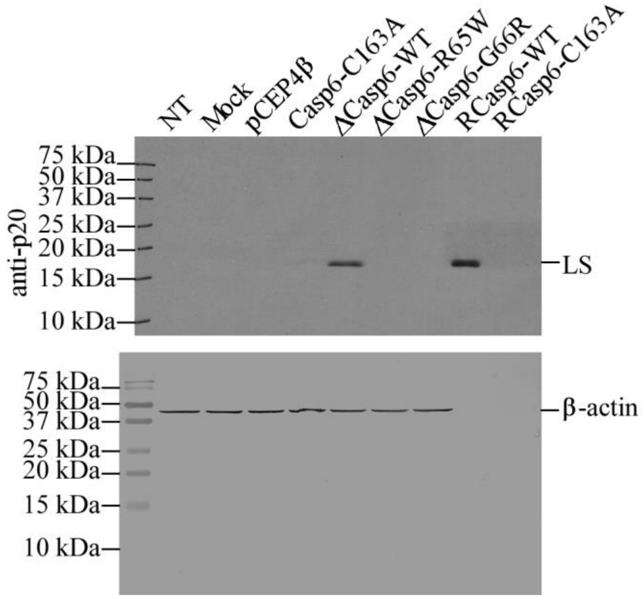

Figure S11. Full-length blots of figure 5c.

Figure 5e

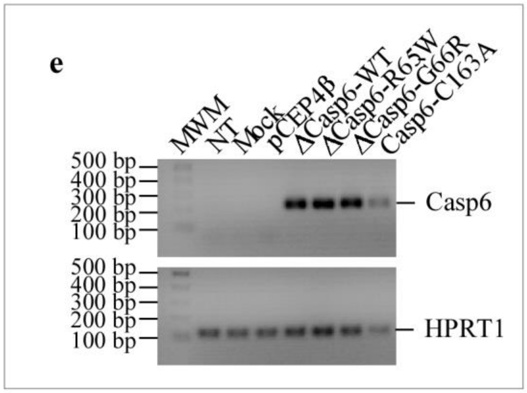

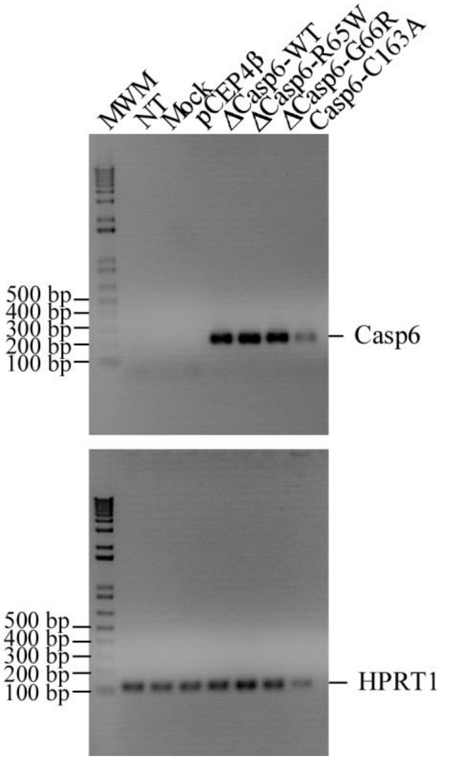

Figure S12. Full-length blots of figure 5e.
